# MIST: Accurate and Scalable Microscopy Image Stitching Tool with Stage Modeling and Error Minimization

**DOI:** 10.1038/s41598-017-04567-y

**Published:** 2017-07-10

**Authors:** Joe Chalfoun, Michael Majurski, Tim Blattner, Kiran Bhadriraju, Walid Keyrouz, Peter Bajcsy, Mary Brady

**Affiliations:** 1 Information Technology Laboratory, National Institute of Standards and Technology, 100 Bureau Dr, Gaithersburg, 20878 MD USA; 2 Physical Measurement Laboratory, National Institute of Standards and Technology, 100 Bureau Drive, Gaithersburg, 20899 MD USA; 3 Fischell Department of Bioengineering, University of Maryland at College Park, College Park, 20742 MD USA

## Abstract

Automated microscopy can image specimens larger than the microscope’s field of view (FOV) by stitching overlapping image tiles. It also enables time-lapse studies of entire cell cultures in multiple imaging modalities. We created MIST (Microscopy Image Stitching Tool) for rapid and accurate stitching of large 2D time-lapse mosaics. MIST estimates the mechanical stage model parameters (actuator backlash, and stage repeatability ‘*r*’) from computed pairwise translations and then minimizes stitching errors by optimizing the translations within a (4*r*)^2^ square area. MIST has a performance-oriented implementation utilizing multicore hybrid CPU/GPU computing resources, which can process terabytes of time-lapse multi-channel mosaics 15 to 100 times faster than existing tools. We created 15 reference datasets to quantify MIST’s stitching accuracy. The datasets consist of three preparations of stem cell colonies seeded at low density and imaged with varying overlap (10 to 50%). The location and size of 1150 colonies are measured to quantify stitching accuracy. MIST generated stitched images with an average centroid distance error that is less than 2% of a FOV. The sources of these errors include mechanical uncertainties, specimen photobleaching, segmentation, and stitching inaccuracies. MIST produced higher stitching accuracy than three open-source tools. MIST is available in ImageJ at isg.nist.gov.

## Introduction

Microscopy imaging of cell cultures must address the spatial scale mismatch between the microscope’s FOV (given its resolution and magnification) and the size of the specimen under study. For example, the area of a standard 6-well plate well is approximately 1000 times larger than the FOV acquired with a 10X objective. Automated microscopy overcomes this limitation by acquiring a grid of partially overlapping images that are stitched together into a time-sequence of composite images. A primary motivation for large coverage imaging is better statistical sampling and the capture of rare events. The probability of observing interesting events increases with respect to the spatial extent and temporal length of the acquisition. These spatial and temporal scales pose challenges for image stitching software. Creating a large mosaic is challenging because the stitching algorithm is sensitive to image feature sparsity in the overlapping regions of adjacent tiles (e.g., during the early period of cell colony growth) and in the computational resources needed for assembling the resulting mosaic. Furthermore, quantitative measurements, such as counts, densities, texture, and protein expressions, depend on the accuracy of stitching algorithms. Existing 2D stitching tools did not meet the accuracy or the runtime requirements to analyze our large dataset projects, like the Oct4 temporal gene expression analysis in stem cell colonies^1^, and some existing 3D stitching tools^2^ are not even applicable to 2D stitching problems.

We address the stitching problem of creating time-lapse mosaics from large 2D grids of overlapping images to enable time-lapse studies of entire cell cultures in multiple imaging modalities. Each tile has a vertical and horizontal overlap with its neighbors. There are three steps for stitching a grid of image tiles: (1) compute candidate translations between adjacent tiles, (2) adjust translations to reduce errors in the stitched image, and (3) compose tiles to produce the mosaic image.

There are two commonly used general approaches to compute the translations between adjacent tiles, feature-based^3–8^ and Fourier-based^2, 9–14^. Feature-based approaches identify matching features in adjacent images and then use these features to compute image translations. However, these techniques require a feature extraction step to detect common features of interest present in two adjacent images. Approaches based on Fourier transforms use image frequency components as the main features. This approach assumes that images have enough pixels with unique frequency components in the overlapping image areas. Both approaches can be effective; the choice of which one to use depends on the overall image content and the characteristics of matching features. Due to its simplicity and predictable parallelism, we decided to include the Fourier-based translation computation.

For the translation improvement step, microscopy imaging of sparsely populated cell cultures generates feature-poor images (e.g., imaging live colonies with low colony density) that introduce errors in the translation computation. A global optimization of the computed translations will reduce the errors in the stitched image. Multiple different optimization techniques can be used to perform this translation optimization: Levenberg-Marquardt^3^ or weighted least squares^9, 15^ applied to all translations, maximize the number of features found in the overlap areas^5^, minimize an error function between hypothesized and actual point correspondences using a joint registration algorithm^8^, maximize the normalized cross correlation^2^, minimize a predefined energy function^13^, or use global geometric and radiometric parameter estimation^16^. These optimization methods do not consider the physical system (e.g., the microscope stage) limitations on the search space, which may lead to large residual errors after optimization in sparse experiments. In contrast, MIST estimates the mechanical stage model parameters from the computed translations to limit the optimization search space and achieve better stitching accuracy.

For the mosaic construction step, multiple approaches exist to assemble the mosaic based on the computed translations. Some treat the translations and their corresponding normalized cross correlations as an undirected graph where an algorithm like the minimum spanning tree^5, 15^ is used to assemble the mosaic. Others treat the problem as an over constrained system of linear equations and solve it using least squares methods^9, 12, 14^, iterative square displacement minimization^7^, or singular value decomposition^2^. With our optimization approach, a minimum spanning tree algorithm produced an accurate mosaic.

We present a novel grid stitching technique called Microscopy Image Stitching Tool (MIST) that estimates the mechanical stage model parameters (actuator backlash, stage repeatability ‘*r*’, and camera angle) from computed pairwise translations and then minimizes stitching errors by constraining and optimizing the translations within a square area of (4*r*)^2^. This constraint reduces the maximum error related to the translation computation for any pair of images. The stage modeling and error minimization methodology is applicable to microscopy images or any mechanical instrument that acquires a 2D grid of image tiles. Another novel aspect of MIST is its multicore hybrid CPU/GPU implementation which can process terabytes of 2D time-lapse multi-channel mosaics 15 to 100 times faster than existing tools. We quantify MIST’s accuracy on three preparations of stem cell colonies seeded at low density and imaged with varying spatial overlap (10 to 50%) where the location and size of 1150 colonies are automatically measured on the microscope. MIST generated stitched images with an average colony centroid distance error less than 2% of a FOV and an average colony area error less than 5%. The sources of these errors include mechanical uncertainties, sample photobleaching, segmentation, and the stitching itself. We show that the area error is mainly due to photobleaching and not stitching, see Supplementary Document section 1 for details. MIST produced the most accurate stitching result when compared to three open-source tools used in the literature on the reference datasets.

## Results

### MIST highlights

**Figure 1:**
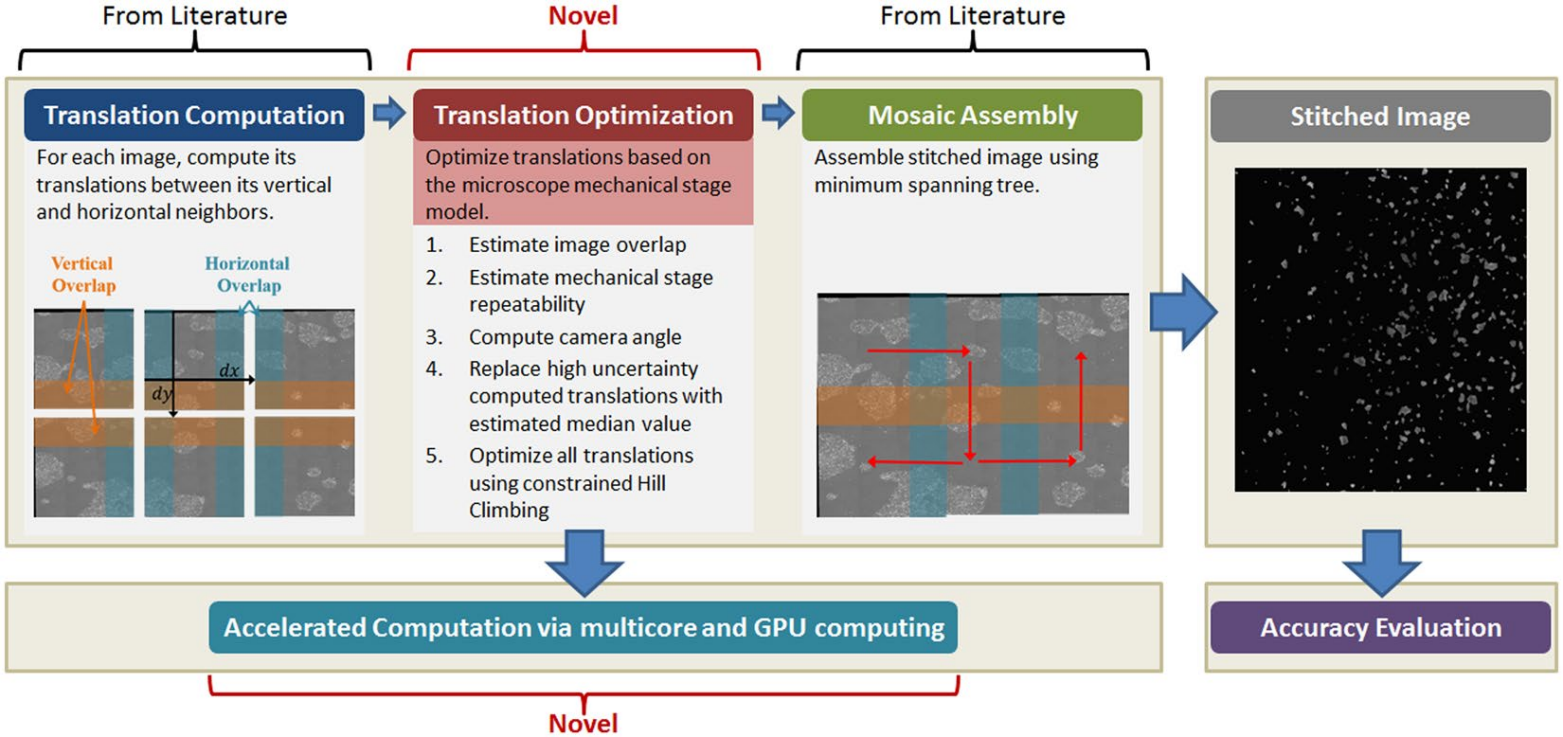
Schematic description of MIST’s algorithm summary and novelties.

Figure 1 highlights MIST’s algorithm and novel aspects: the global optimization and the performance.

### Application across image content, modalities, and instruments

**Figure 2:**
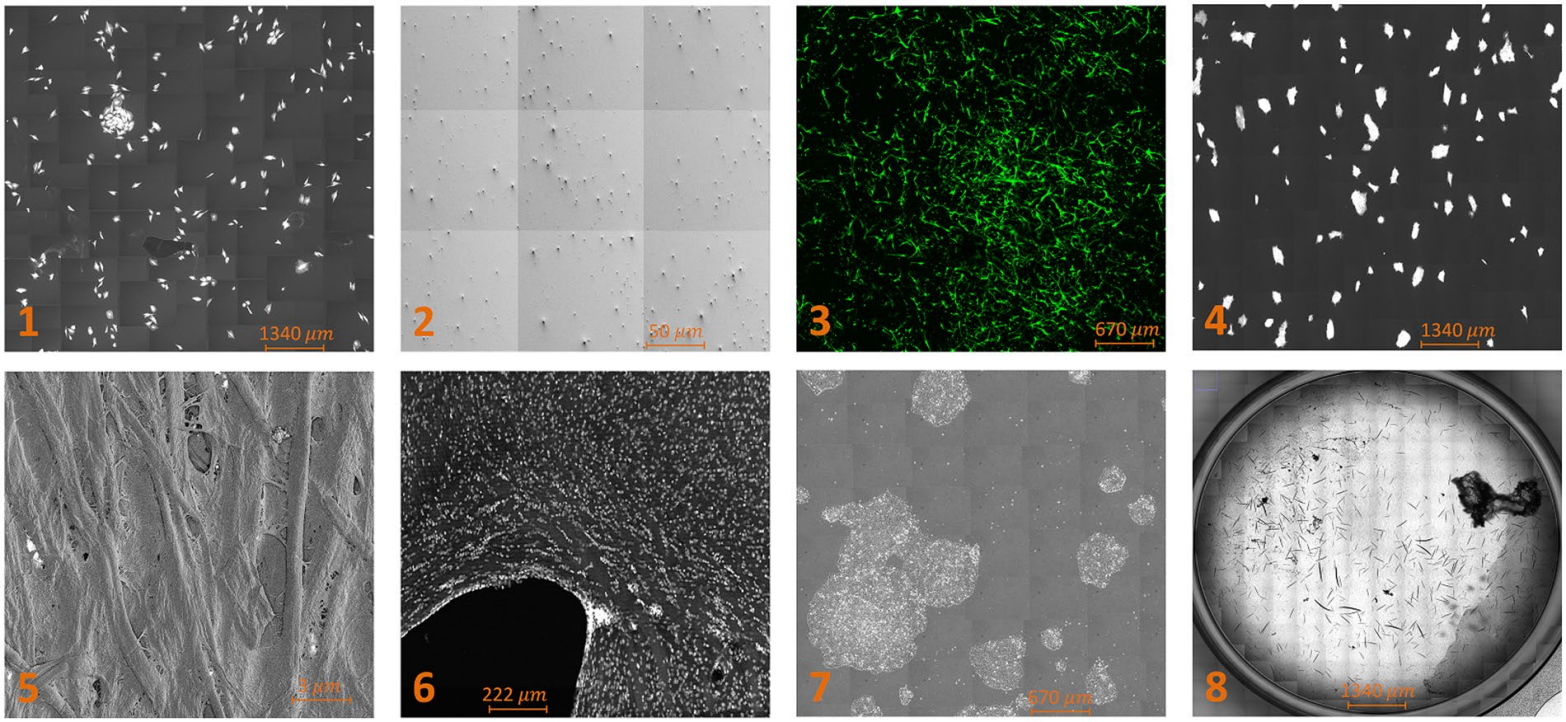
MIST application example images: (**1**) A10 cells, (**2**) Carbon Nanotubes, (**3**) HBMSC, (**4**) IPS cell colonies, (**5**) Paper nanoparticle, (**6**) Rat brain cells, (**7**) Stem cell colonies, and (**8**) Worms.

We evaluated MIST’s applicability and robustness on over a thousand fully stitched images (Fig. 2). These large and diverse datasets comprise multiple image content types, imaging modalities, microscopes, and data sizes. They were acquired using five microscopes (Leica, Olympus, Nikon, Focused Ion Beam Scanning Electron Microscope (FIB-SEM), and Zeiss), four imaging modalities (fluorescent, phase contrast, bright-field, and FIB-SEM), eight content types (A10 cells, carbon nanotubes, Human Bone Marrow Stromal Cells (HBMSC), Induced Pluripotent Stem Cells (IPSCs), paper nanoparticles, rat brain cells, Human Embryonic Stem Cells, and C. elegans), large range of image overlaps (10–70%), and a large range of grid sizes (5 × 5 to 70 × 93). These datasets are available at isg.nist.gov.

### Quantitative accuracy measurement

Accuracy metrics: we use the following three metrics to quantify the stitching accuracy when comparing reference and stitched measurements over regions of interest (ROI): (1) false positive (added ROIs) & false negative (undetected ROI), (2) centroid distance error in pixels (*D*_*err*_), and (3) percent area error (*S*_*err*_) between ROIs.

We used three regular 6-well plates of stem cell colonies to generate stitching reference and testing datasets. Only one well is used in each plate where stem cell colonies are seeded at low density to allow for colony growth.

Reference dataset: for each plate, we automatically imaged each colony with an exposure time that produces a high quality fluorescent image and centered it inside the FOV by an automated microscopy feedback loop. This loop minimizes the difference between the colony centroid and the middle of the FOV until it reaches convergence (difference less than 1 μm). The final centroid location for a colony is saved from the microscope stage controller and its area measured by segmentation. More detail is available in Supplementary Document section 1.5.

Test dataset: for each plate, we imaged an inscribed square area with five overlap values ranging from 10% to 50% for a total of 5 × 3 stitched test datasets.

We ran MIST and three other open-source tools on the test datasets and generated the corresponding stitched images. These tools: FijiIS^9^, TeraStitcher^15^, iStitch^17^, had implementations available from the literature and operate on 2D images. We measured stitching accuracy using the three metrics *FP* + *FN*, *D*_*err*_, and *S*_*err*_. Table 1 summarizes the stitching accuracy quantitative measurement across all three reference datasets. The results were combined on all three experiments by adding the *FP* + *FN* metric and by reporting the average and standard deviation of the *D*_*err*_, and *S*_*err*_ metrics.

**Table 1:** Stitching accuracy measurement results across all three experiments.

Metric	Tool	10% overlap	20% overlap	30% overlap	40% overlap	50% overlap
*FP* + *FN* (Count)	MIST	1	1	1	2	2
	TeraStitcher	2	8	6	13	5
	iStitch	1008	1052	960	574	748
	FijiIS	3	2	4	4	5
*D*_*err*_ (Pixels)	MIST	14 ± 7	16 ± 8	16 ± 9	16 ± 8	16 ± 8
	TeraStitcher	18 ± 9	16 ± 10	19 ± 11	19 ± 9	18 ± 10
	iStitch	153 ± 0	679 ± 0	586 ± 87	164 ± 80	254 ± 122
	FijiIS	14 ± 44	29 ± 52	15 ± 8	15 ± 7	29 ± 40
*S*_*err*_ (Percent)	MIST	−1 ± 4	1 ± 5	0 ± 5	1 ± 5	0 ± 5
	TeraStitcher	−1 ± 5	3 ± 6	2 ± 7	5 ± 8	5 ± 8
	iStitch	−14 ± 0	−59 ± 0	1 ± 8	3 ± 5	0 ± 7
	FijiIS	−1 ± 4	1 ± 5	0 ± 5	1 ± 5	0 ± 5

It is important for any stitching tool to minimize all three metrics, but paramount is the *FP* + *FN* metric, which measures ROI duplication or deletion. The *FP* + *FN* metric should have a value as close as possible to zero for any accurate stitching. The results show that MIST has the lowest *FP* + *FN* metric among all evaluated tools. Any colony with a centroid distance error larger than the smallest dimension of the FOV is considered missed, thereby increasing the value of the *FP* + *FN* metric. The *D*_*err*_ metric measures the global accuracy of a stitching method over the entire reconstructed image. MIST has consistently one of the lowest average and standard deviation *D*_*err*_. The *S*_*err*_ metric measures the local stitching accuracy over an ROI (in this case a colony). Both MIST and FijiIS performed well locally over the colonies area. TeraStitcher did well on the low overlap datasets but not as well on the higher overlaps. iStitch did not produce any results for all the test datasets.

### Qualitative accuracy measurement

For our application, feature sparse image grids occur at the early time-points of a time-lapse experiment where colonies are seeded at low density to allow for growth^1^. Time-lapse stem cell colony experiments often start with very low colony seeding densities to give the colonies room to grow. The test dataset for qualitative accuracy measurement is our Stem Cell Replicate1^1^. Each grid of the 161 phase-contrast time-lapse images consists of 18 × 22 images (396 total) acquired at 10% overlap.

Due to the lack of reference measurements in these large-coverage time-lapse experiments with live stem cells we resorted to visual inspection and a qualitative distance error metric to measure the general correctness of the stitched images.

**Figure 3:**
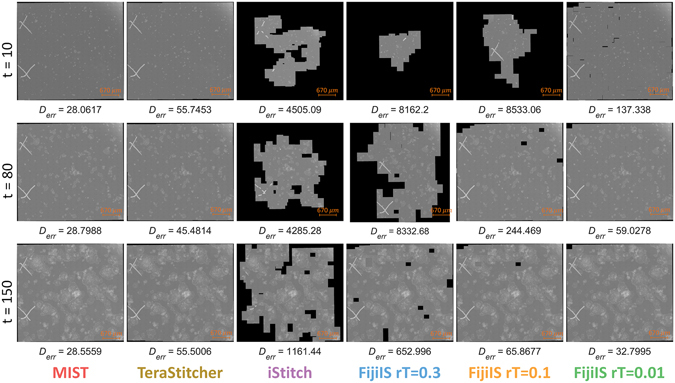
Example of stem cell colonies over time that are stitched by all four tools and with different values of the regression threshold in FijiIS.

**Figure 4:**
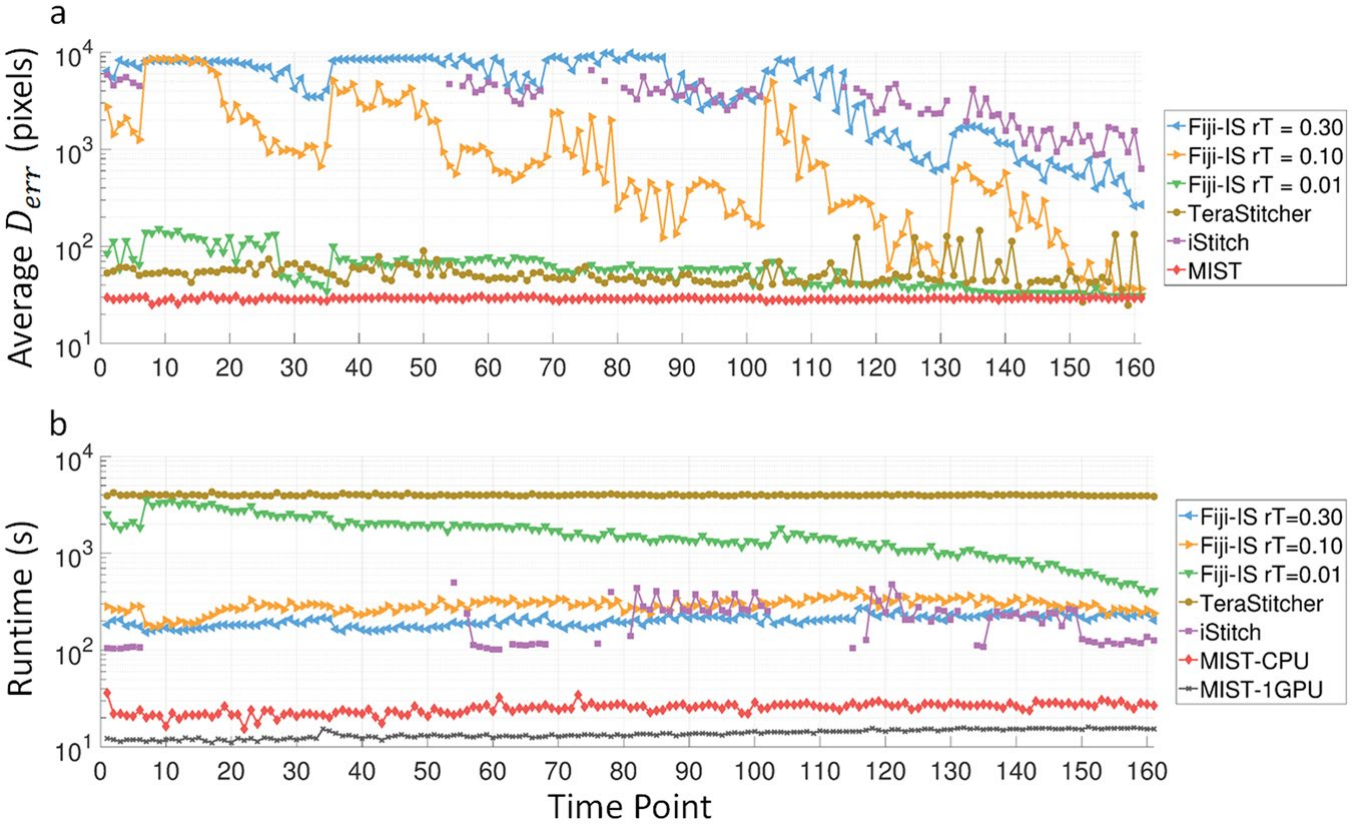
(**a**) Qualitative stitching accuracy and (**b**) Execution time throughout the time-sequence.

This qualitative stitching accuracy illustrates the robustness of MIST in stitching large feature-sparse datasets. Figure 3 shows three example images of each tool for different time points and Fig. 4 displays the distance error to the reference 10% overlap image grid and the corresponding runtime for each tool.

All three open-source tools (TeraStitcher, iStitch, and FijiIS) failed to produce a correctly stitched image at some point in the time-sequence. The final stitched images should not have any blank pixels inside the gridded boundaries because the 10% overlap between FOVs (67 *μm*) is large enough to overcome any actuator backlash or stage position errors. A blank pixel in grayscale images is defined as a pixel that is neither a background nor a foreground and has no intensity value ( = 0). Figure 3 shows blank pixels cutting through the colonies for the TeraStitcher assembled image at *t* = 150. TeraStitcher performed well for the early time points, but was challenged by the later time points. Figure 4 illustrates stitching errors for TeraStitcher at late time-points (*t* = [125,140] and *t* = [155,161]) similar to the example shown (*t* = [150] in Fig. 3). This tool’s slow runtime (multiple hours per image) prevents its use on such large datasets. iStitch was unable to produce viable results for any time point, in some cases it had not finished after 10^4^ s. FijiIS has a regression threshold (*rT*) parameter, which when lowered improved the accuracy of the stitched image at the cost of longer runtimes. The default *rT* value (0.3) does not yield a viable result for any time-point. Even with a very low regression threshold, FijiIS does not produce reliable stitched images during the early time points. It does well at later time points where colonies are big enough to cover the overlapping area between consecutive images. However, for such a low regression threshold value, FijiIS is as slow as TeraStitcher during the early time points.

### Performance

The MIST ImageJ plugin was developed with performance in mind so it can process large datasets in a timely manner especially with respect to time-lapse studies. Our specific performance goal is to stitch large grids (55 × 55 images of 1040 × 1392 pixels each) in less than 1 minute, thereby allowing enough computing time to analyze the acquired images before the start of the next imaging cycle. Our strategy to achieve this goal is to take advantage of the hardware parallelism that is available in a single machine with multicore CPUs and GPUs. As an illustration of MIST’s temporal scalability, MIST stitches all 161 time points of the feature-sparse Stem Cell Replicate1 dataset (Fig. 4) in 67.2 minutes on our test system (with two high end Intel Xeon CPUs). In contrast, FijiIS (*rT* = 0.01) requires 73.7 hours using the same hardware (nearly 65 × slower). Furthermore, adding one NVIDIA Tesla K40 GPU to the test machine nearly halves the dataset’s processing time, down to 36.6 minutes (1.8 × speedup). Individual time points stitch at interactive rates, requiring an average of 25 and 14 seconds for the CPU and GPU, respectively. This allows users to interact with their data in near real-time. In general, adding a second GPU can reduce the execution time for large grids; however, this is not the case for the 18 × 22 grids in this dataset as processing with two GPUs becomes dominated by disk I/O.

**Figure 5:**
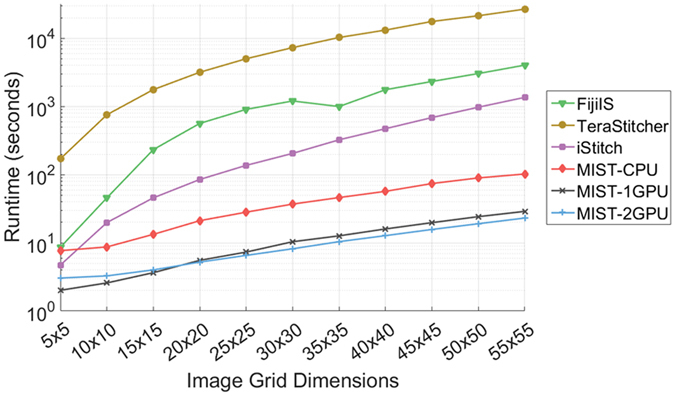
Stitching execution times for varying grid sizes (number of images).

Figure 5 illustrates the scalability of all 4 stitching tools for successively larger image grids. To reduce potential confounding factors, each grid is a subset of a 55 × 55 tile dataset consisting of phase contrast images of stem cell colonies acquired with a 10× objective covering a 10 cm well with 10% image overlap (50 000 × 70 000 pixel stitched image). By sub-setting a single dataset, both image content and overlap are constant while the grid size varies. The reported times are an average of 10 consecutive runs with the 10^th^ and 90^th^ percentiles removed. MIST-CPU outperforms the other stitching tools by 1–3 orders of magnitude and scales better as the image grid increases in size. Additionally, MIST-1GPU is on average 3.6× faster than MIST-CPU with identical scaling. This speedup improves upon the 1.8× speedup from the temporal experiment, which is limited by the Stem Cell Replicate1 image data being stored on a standard hard drive versus this dataset being cached in memory. This factor highlights the effect that disk I/O has on scalable performance. Adding a second GPU improves performance for grids larger than 25 × 25; providing an average 4.5× speedup compared to MIST-CPU. With grids smaller than 20 × 20 MIST-2GPU is slower than the 1 GPU version due to setup overhead, GPU-to-GPU communication, and there not being enough work to keep both GPUs busy.

## Discussion

MIST is a robust 2D time-lapse multi-channel stitching tool that can be used on any grid-based dataset like those acquired by a microscope. Its demonstrated accuracy, performance, and scalability make MIST optimal for handling large microscopy datasets. MIST is available in MATLAB and as an ImageJ/Fiji plugin with CPU and GPU implementations from isg.nist.gov.

MIST has several limitations that should be noted. It was designed to stitch microscopy images acquired using a mechanical stage which moves the sample in a repeatable grid pattern. Additionally, MIST expects the overlap error between images to be less than a pre-defined actuator backlash (default value of ±3%). Any computed overlap value beyond that error is deemed unreliable. This stage mechanical model prevents MIST from handling stitching problems with varying overlaps within the same acquisition. MIST is designed to perform 2D time-lapse multi-channel stitching. It cannot perform volumetric (3D) stitching, but MIST’s ability to stitch a time sequence makes it usable for 3D data if the z-stack is acquired for every (*x*,*y*) location as explained in Supplementary Document section 3.2. MIST uses normalized cross correlations (*ncc*) to compute image registrations. The presence of high levels of image noise can affect this metric and impact stitching accuracy. If the images being stitched are very noisy, then they may need to be preprocessed before stitching. Preprocessing images with a Gaussian filter, for example, can reduce the noise level and smooth the derived *ncc* surface. Supplementary Document section 3.3 presents an example of preprocessing noisy carbon-nanotubes images.

## Methods

Regular stitching algorithms typically consist of three steps: (1) compute candidate translations between adjacent tiles, (2) optimize translations to reduce stitching errors in the mosaic image, and (3) produce the mosaic image based on the translations.

Our algorithm introduces an estimation of the microscope mechanical stage parameters as a new step between the steps 1 and 2 if the stage parameters are not provided by the user. MIST uses the stage modeling to perform translation optimization. Translation optimization is needed in experiments with intensity-homogeneous background and sparse foreground objects because of poor signal in overlapping areas. This causes large errors in the estimated tile translations. In addition, translations have a large uncertainty in the relative stage position due to the stage repeatability, that amounts to 1 to 2 microns for good microscopes, and the microscope’s camera angle between the stage coordinate system and the camera coordinate system. The user might specify a desired 10% spatial overlap between consecutive tiles but the actual overlap value could fluctuate between 8% and 12%. This fluctuation is the reason why naïve stitching with fixed overlap will not be accurate. Our method takes advantage of the stage modeling to limit the search space around the 10% value within a range that is proportional to the amount of estimated (or given) stage repeatability.

The MIST algorithm consists of the following four steps: (1) compute candidate translations between adjacent tiles, (2) estimate mechanical stage model from computed translations if model parameters are not given by the user, (3) optimize translations to reduce stitching errors in the mosaic image, and (4) compose tiles to produce the mosaic image.

### Translation computation

MIST uses a Fourier-based approach for simplicity and predictable performance since it does not need an additional feature detection tool. MIST implements the Phase Correlation Method^18^ (PCM) to compute translations between adjacent tiles. PCM is based on the Fourier Shift Theorem which computes the spatial shift between two images as a phase shift in the frequency domain.

In real images, phase correlation (*PC*) contains several peaks that correspond to different translation values^9^. To determine the correct translation, the top two peaks in the *PC* matrix are evaluated. The number of peaks is adjustable and the default is two. Due to the periodicity of the Fourier domain, each peak corresponds to four different possible translations (in 2D). We evaluate these four possible translations, for each peak, using the normalized cross-correlation (*ncc*) of the overlap area between adjacent images. The candidate translation with the highest *ncc* value is selected as the translation between two adjacent images.

### Estimation of mechanical stage model from pairwise translations

There is a degree of uncertainty in the translation computation that causes errors in the stitching results. The sources of errors that affect translation computation between pairs of images are: (1) the signal to noise ratio in the acquired image, (2) the amount of signal in the overlap area, (3) the signal distribution with respect to the stage movement in the overlap area (i.e., flat-field effect in some imaging modalities, uniform or periodic signal distribution), and (4) the mechanical imperfections of the automated microscope stage (i.e., stage repeatability and actuator backlash).

Moreover, the mechanical stage model parameters vary over time and the variation magnitude depends on the microscope usage. If the microscope’s user calibrated the equipment and measured the stage repeatability, there is an option to input such parameters in the advanced tab of the tool. If the user inputs those parameters, then MIST does not estimate them. However, it is difficult and time consuming to calibrate and estimate the mechanical stage properties of the microscope.

Additionally, there are research environments where a microscope might have multiple users. Each user might adjust/change/perturb the camera settings or mechanical stage. These physical changes can alter the microscope to be out of calibration.

Finally, there are time-lapse experiments in which touching the mechanical stage cannot be avoided. For example, cells in live experiments need to be fed intermittently. To do this, the sample is removed from the microscope and then put back on the stage after media change. This feeding process can alter the mechanical stage properties among many other experimental settings.

Therefore, we are offering an automated way to estimate these stage parameters from the computed translations to prevent the user from having to calibrate and measure these parameters before every acquisition.

**Figure 6:**
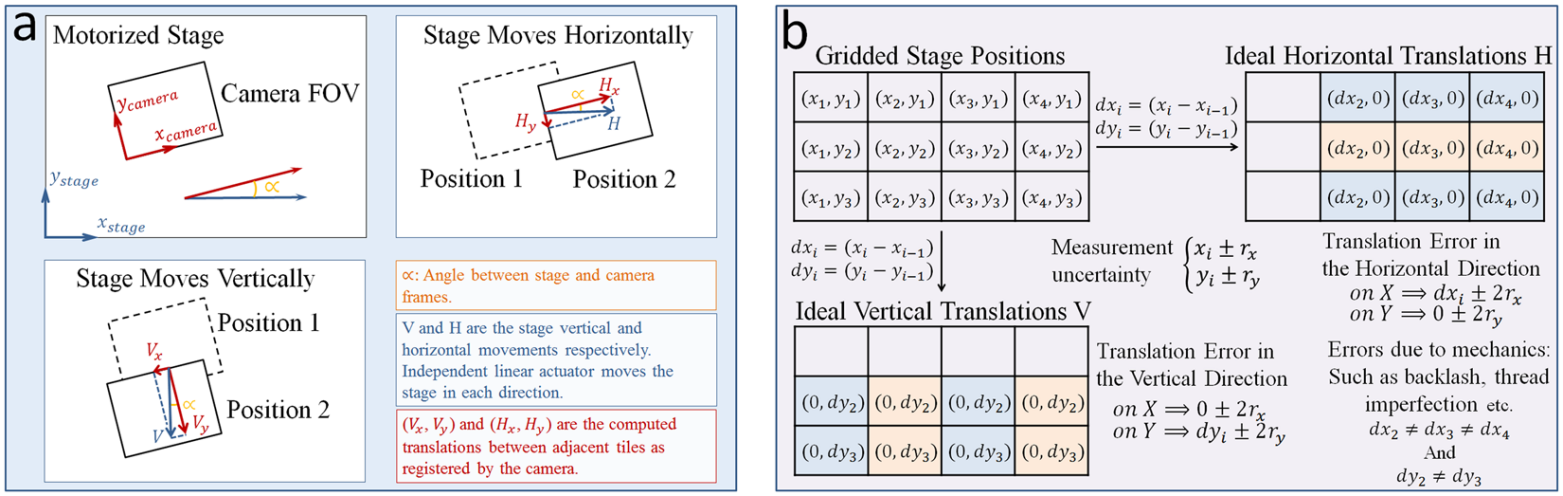
Stage mechanical model. (**a**) Stage displacements as observed by the camera. (**b**) Uncertainty and errors of horizontal and vertical tile translations due to stage mechanical properties.

An automated microscope has two co-planar coordinate frames, the observation frame (i.e., camera) and the control frame (i.e., stage actuators), that are related by the camera angle *α*, as shown in Fig. 6.a. This angle is difficult to calibrate. Therefore, a misalignment between the camera and stage axes will remain in most experiments. The camera observes the horizontal and vertical stage movements, *H* and *V*, as (*H*_*x*_,*H*_*y*_) and (*V*_*x*_,*V*_*y*_) which are computed as follows:

1 $$[\begin{array}{c}{H}_{x}\\ {H}_{y}\end{array}]=[\begin{array}{c}H\,\cos \,\alpha \\ -H\,\sin \,\alpha \end{array}]\,{\&}\,[\begin{array}{c}{V}_{x}\\ {V}_{y}\end{array}]=[\begin{array}{c}V\,\sin \,\alpha \\ V\,\cos \,\alpha \end{array}]$$

A motorized mechanical XY-Stage moves a biological sample relative to the microscope’s optical column. This movement is carried out by two independent stepper motor linear actuators, one for each direction. The mechanical uncertainty of such a system is known as the stage repeatability. Moreover, the imperfection of the stage as a mechanical device introduces a variable overlap between adjacent tiles. Modeling the mechanical properties of a stage provides an upper bound to the variable overlap and can be used to limit the search for optimal translations, thereby minimizing the margin of stitching error.

Figure 6.b shows a grid tiling with the positions (*x*,*y*) that the stage will visit. Each position has an uncertainty equal to the stage repeatability (*x* ± *r*_*x*_,*y* ± *r*_*y*_). However, translations (*dx*,*dy*) computed in the vertical or horizontal directions between adjacent tiles are differences between respective positions. Therefore, the maximum possible error in the computed translation values is (*dx* ± 2*r*_*x*_,*dy* ± 2*r*_*y*_).

The horizontal and vertical translations in the image coordinate system must account for the camera angle as well as the mechanical uncertainties. The equations for horizontal and vertical translations that include the microscope models are the following:

2 $$\{\begin{array}{c}{H}_{x}=d{x}_{i}\,\cos (\alpha )\pm 2{r}_{x}\\ {H}_{y}=d{x}_{i}\,\sin (\alpha )\pm 2{r}_{y}\end{array}\,{\rm{and}}\,\{\begin{array}{c}{V}_{x}=d{y}_{i}\,\sin (\alpha )\pm 2{r}_{x}\\ {V}_{y}=d{y}_{i}\,\cos (\alpha )\pm 2{r}_{y}\end{array}$$

MIST estimates the following four quantities from the translation matrices *H* and *V*: (1) overlap amount, (2) the camera angle, (3) the microscope stage repeatability, and (4) the microscope backlash. Supplementary Document section 5 describes the details of this estimation.

### Translation optimization constrained by stage repeatability

Figure 6.b shows each column in *H* as having the same *dx*_*i*_ and the same *dy*_*i*_ for each row in *V* within a ±2*r* limit. *dx*_*i*_ values differ between the columns of *H* while *dy*_*i*_ values are different between the rows of *V* due to backlash and mechanical imperfections. As such, we filter *H* column wise and *V* row wise where we replace all computed translations, whose *dx*_*i*_ or *dy*_*i*_ values deviate from the median value by more than 4 × *r* (the stage repeatability), by the median value in that direction. We then apply Constrained Hill Climbing^19^ to the *ncc* values centered at the median translation and constrained within 4 × *r* region. Hill climbing will find the translation with the maximum *ncc* value by following the steepest gradient. The 4 × *r* constrain comes from the model and bounds the algorithm to a small search space while converging to an optimal value.

### Mosaic assembly

The assembly problem can be represented as an undirected graph where vertices are tiles and ncc values are edges. Each tile is connected to its surrounding four neighbors, three neighbors for tiles on edges and two neighbors for tiles on corners. This over-constraint problem needs to be resolved to construct a well-formed image. We use the weighted maximum spanning tree algorithm^20^ to find the optimal subset of edges that connects all tiles together, without any circular subsets of edges per tile (each tile is connected only once to the reconstructed image) while maximizing the sum of all weights along that path. The weight of all computed translations that satisfy the physically plausible offset stage model criteria (offsets <4*r*) are increased to ensure preferential selection of these translations during assembly. When creating the final stitched image, a linear or non-linear blending is applied to each individual tile^12^ to compensate for shading differences in overlapping areas between adjacent tiles. Supplementary Document section 6 gives more detail about the mosaic assembly process.

### Accuracy measurement

The mathematical formulas of the two performance metrics are:

3 $${({D}_{err})}_{i}=\sqrt{{({x}_{{m}_{i}}-{x}_{{c}_{i}})}^{2}+{({y}_{{m}_{i}}-{y}_{{c}_{i}})}^{2}}$$

4 $${({S}_{err})}_{i}=\frac{{A}_{{c}_{i}}-{A}_{{m}_{i}}}{{A}_{{m}_{i}}}\times 100$$

where *D*_*err*_ is the centroid distance error metric, $({x}_{{m}_{i}},{y}_{{m}_{i}})$ is the measured centroid location of reference colony *i* and $({x}_{{c}_{i}},{y}_{{c}_{i}})$ is its computed centroid location from the stitched image. *S*_*err*_ is the size error metric in percent, ${A}_{{m}_{i}}$ is the measured area in pixels of reference colony *i* and ${A}_{{c}_{i}}$ is the computed area from the stitched image and *i* = 1,…,*N* is the number of reference colonies.

The reference measurements are single colony images where the colony is automatically centered in the middle of the FOV and the corresponding stage coordinate locations (*x*_*r*_,*y*_*r*_) are saved in a CSV file. The output of a stitching method is a mosaic image from which colonies are segmented, cropped, and their locations computed. This will generate one image per centered colony. We then use the Hungarian algorithm^21^ to find the optimal matching pairs between the two datasets. We apply the Hungarian algorithm to the similarity matrix *T* based on the *ncc* value between all reference colonies and the stitched ones. The output of a stitching method may result in adding some colonies by duplication or deleting some colonies by misaligning tiles. This results in a difference between the reference and stitched colony counts. This difference is characterized by the *FP* + *FN* metric. Furthermore, FN and FP metrics increase in value when the centroid distance of two matching colonies is larger than half a field of view (e.g., 340 μm). The corresponding match between such colonies is deleted; the reference colony will be considered as missed and the corresponding stitched colony as added.

We adjust the computed locations from the stitched image for rotation and translation due to the camera angle between absolute position read on the microscope controller and the relative positions computed from the stitched image. We use Kabsch’s algorithm^22^ to compute the optimal rotation matrix that minimizes the root mean squared deviation between the two sets. Supplementary Document section 1 gives more detail about this methodology.

### Data acquisition protocols

The H9 human embryonic stem cell line (WiCell, Madison, WI)^23^ was prepared to express GFP under the influence of the native Oct4 promoter and images were collected as previously described^24^. This study was approved by NIST and carried out in accordance with the approved guidelines of the NIST Human Subjects Protection Office. The final dataset consists of three data collections, each collection with around 161 time-lapse images at roughly 20 000 × 20 000 pixels per stitched image frame.

### Implementation

The MIST ImageJ plugin is a parallel application that uses coarse-grained parallelism to take advantage of computing resources available in a high-end workstation, namely multi-core CPUs and zero or more GPUs^25^. The salient features of MIST’s implementation are: (1) its pipelined workflow architecture, (2) its use of GPUs, and (3) its memory management. The pipelined workflow architecture organizes the image stitching algorithm as a series of connected stages that operate concurrently on multi-core CPUs. Data flows from one stage to the next using shared thread-safe first in, first out queues. The processing stages within image stitching operate on either computational, input/output (I/O), or state update tasks. For example, one computational stage might perform Fourier transforms, while an I/O stage reads images, and a bookkeeper determines when to issue more work. This creates a separation of concerns, disconnecting algorithm state management from the individual computational elements the algorithm is composed of. This allows computational stages to begin processing without considering the overall state of the computation, which improves CPU utilization. A computational stage is configured to operate using multiple workers (threads); this design improves the stage’s data production and consumption rates. In addition, I/O stages and computational stages operate concurrently, which allows for data motion to overlap with computation (such as overlapping reading images from disk with computing the FFTs of other images).

This workflow system is also used to take advantage of GPUs by altering the computational stages to issue work for GPU accelerators, preceded by a copy between the address space of the CPUs and GPUs. Furthermore, MIST instantiates one GPU-based pipeline workflow per GPU and distributes data across all pipelines, thereby allowing it to take advantage of multiple GPUs within a single machine.

MIST diligently manages memory and reclaims buffers so it only needs to keep one row (or column) of image tiles in memory. It does so by allocating all required memory buffers at startup and then recycling buffers as it completes the processing of individual image tiles. This management is important to enable the CPU-oriented implementation to process large datasets: the Fourier transform that corresponds to a tile or an adjacent tile pair is 8 or 16 times the size of the tile itself; keeping all the tiles and transforms in memory quickly uses up all the available physical memory in a machine and results in very poor performance. This management of memory is even more critical when using GPUs because memory is a much scarcer resource on GPUs; even high-end GPUs have far less memory than comparable CPUs. See Supplementary Document section 2 for more details.

### Hardware

All runtimes discussed were generated using Ubuntu 14.04.4 LTS (Linux kernel 3.19.0–68, OpenJDK IcedTea 2.6.7, and FFTW 3.3^26^) with 2× Intel® Xeon® E5–2650 v3 CPUs @2.30 GHz, 128GB DDR4 memory, 2× NVIDIA Tesla K40 GPUs (CUDA 7.5^27^).

### Disclaimer

The identification of any commercial product or trade name does not imply endorsement or recommendation by the National Institute of Standards and Technology, nor is it intended to imply that the materials or equipment identified are necessarily the best available for the purpose.

## Electronic supplementary material

Supplementary Document
