# Supplementary material for: MIST: Accurate and Scalable Microscopy Image Stitching Tool with Stage Modeling and Error Minimization
Source: Sci Rep. 2017 Jul 10;7:4988. doi: 10.1038/s41598-017-04567-y (PMC5504007; doi:10.1038/s41598-017-04567-y)
Supplement: Supplementary file 1 — Supplementary Document [file 41598_2017_4567_MOESM1_ESM.pdf]

MIST: Accurate and Scalable Microscopy Image Stitching Tool with Stage Modeling and Error Minimization Supplementary Document

Joe Chalfoun[1,*], Michael Majurski[1], Tim Blattner[1], Kiran Bhadriraju[2,3], Walid Keyrouz[1], Peter Bajcsy[1], and Mary Brady[1]

Contents

0 Introduction 2
1 Evaluation Methodology 2
1.1 Overview 2
1.2 Reference Image Content 2
1.3 Image Segmentation 3
1.4 Generate Reference Dataset 3
1.5 Generate test dataset 5
1.6 Evaluate Stitched Images 5
1.7 Stitched Image $Derr$ Heat Maps 8
2 Implementation and Performance 9
2.1 Demonstration Datasets 10
2.2 Application Details 12
2.3 Time-Lapse Application Dataset Performance 12
3 MIST Stitching Limitations 13
3.1 A Regular Grid is Required 13
3.2 Limited to 2D+time+channel Image Sequences 14
3.3 Noisy Images Might Require Preprocessing 14
4 XY-Stage Actuator Movement Model 17
5 Stage Model Parameter Estimation 18
6 Tilt and plate movement Assessment 19
7 Image Composition 19
8 References 20

0 Introduction

This document describes the technical detail about MIST algorithm and implementation.

Section 1 is about the evaluation methodology. It has seven subsections that describe image segmentation, the creation of the reference dataset and test datasets, and the evaluation of a stitching result based on the selected metrics.

Section 2 describes MIST implementation and performance measurement.

Section 3 highlights MIST limitation factors and possible ways to overcome those limitations.

Section 4 illustrates the mechanical stage model.

Section 5 depicts the estimation of the mechanical stage model derived from the computed translations.

Section 6 assess the effect of plate tilt and solution movement during acquisition.

Section 7 is about stitched image reconstruction from single tiles after translation optimization.

1 Evaluation Methodology

1.1 Overview

We evaluate the accuracy of an image stitching algorithm in three steps: (1) generate reference data, (2) create test dataset and run the stitching algorithm, and (3) compute the error metrics.

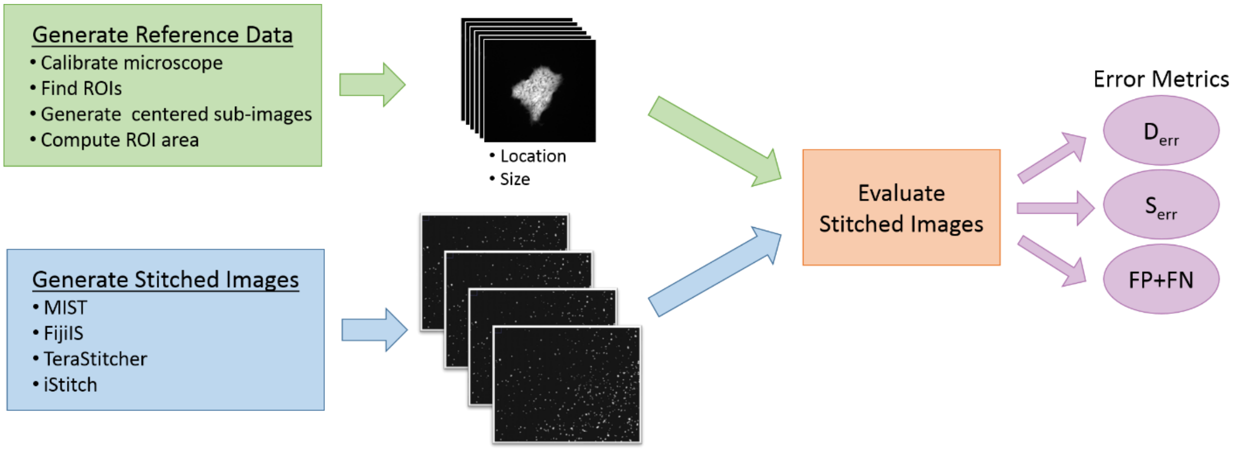

Figure 1: Outline of the image stitching algorithm evaluation methodology.

The reference dataset generation is done in five steps: (1) find relevant regions of interest that fit within one field of view in the experiment, (2) automatically or manually locate them on the microscope stage, (3) acquire high resolution images of these regions, (4) compute their reference areas, and (5) save their corresponding reference locations.

1.2 Reference Image Content

The reference and test datasets consist of three plates of stem cell colonies fixed on days 2, 3, and 4 respectively. We imaged each plate at 10x magnification in the fluorescent modality (Cy5 staining).

1.3 Image Segmentation

An image segmentation algorithm, shown in Figure 2 is required to measure the reference ROI area's and centroid location's. To simplify this requirement, we selected the exposure time to produce high contrast images where the manually selected segmentation threshold of 500 intensity units correctly segments an image. Additionally, we remove objects smaller than 2-3 individual cells by requiring all foreground objects be at least 2000 pixels in area.

Algorithm: Image Segmentation Algorithm

Input: 2D Image I, $threshold$, $minimumArea$
Output: Foreground Mask M

```
begin
    // threshold the input image
    M = I > threshold

    // remove foreground objects smaller than 2000 pixels
    M ← bwareaopen(M, minimumArea)
    return M
end
```

Figure 2: Pseudo-code for image segmentation algorithm.

1.4 Generate Reference Dataset

We generated reference stitching datasets on a calibrated and motorized Olympus microscope stage controlled by MicroManager. MicroManager enabled us to convert the open loop stage controller system into a closed loop using image processing. The reference stitching data consists of high accuracy ROI location and area metadata. To accurately measure the area, each ROI must fit within a single microscope Field of View (FOV). To accurately measure the ROI location, we control the microscope stage via image analysis to place each ROI's centroid in the middle of the acquired reference image. By centering the ROI, we can measure its stage location to within the mechanical stage repeatability. By placing the ROI completely within the FOV, we can measure its area to within the segmentation accuracy. Both measurements are independent of stitching. See Section 1.4.1 for more details on our closed loop stage feedback system.

Once each ROI is centered within the FOV, we acquire a reference image of the colony. Each reference data point consists of the centered colony image, its stage position P_r, and its area. One of the three resulting reference datasets is shown in Figure 3.

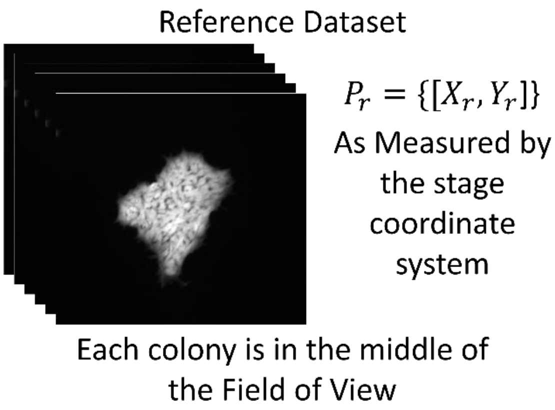

Figure 3: Example reference dataset containing centered colony images and stage positions

1.4.1 Reference Colony Centering

The colony centering algorithm is part of the closed loop microscope control software written for this evaluation. The centering algorithm moves the microscope stage to place the centroid of a colony in the middle of the image FOV. If there is more than one colony in the FOV, the colony with the centroid closest (as defined by Euclidean distance) to the middle of the FOV will be centered.

The centering algorithm acquires an image and saves the current stage location as read by the stage controller. This image is segmented and object centroids are computed with respect to the FOV. The object with the centroid closest to the middle of the FOV is selected, all other objects are deleted from the foreground mask. The distance d (in pixels) between the current colony centroid and the middle of the FOV is computed. That distance is converted to micrometers with the user supplied pixel to micrometer conversion ratio (e.g., 0.658 pixels per micrometer for this experiment). The centering algorithm translates the current stage position to the new position by adding the distance d and moves the stage to the new position. The algorithm repeats until the position is stable ($d < 1\mu m$) or a maximum number of iterations is reached (10 iterations). The colony centering typically converges in 1 to 3 iterations. The colony centering steps are shown in Figure 4.

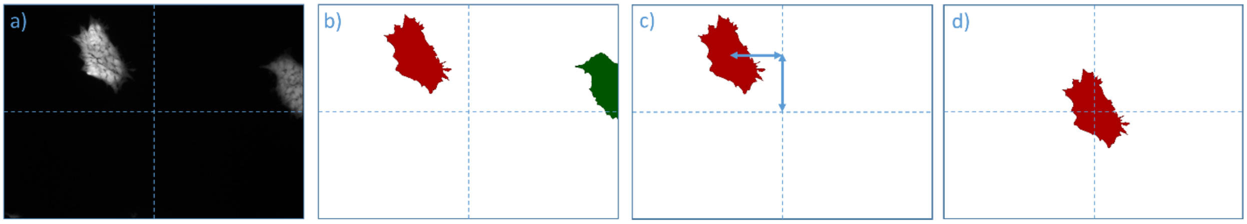

Figure 4: Example colony centering steps: a) initial image, b) segmented and labeled, c) translation delta computed, d) translation delta applied

The algorithm performs iterative refinement to handle colonies that start partially out of the FOV and to correct calibration errors in the stage, pixel to micrometer ratio, and camera angle. For example, if the camera coordinate system is rotated with respect to the stage coordinate system the computed translation delta will not produce the expected colony movement within the FOV. Figure 5 demonstrates the effect of a 10 degree camera rotation.

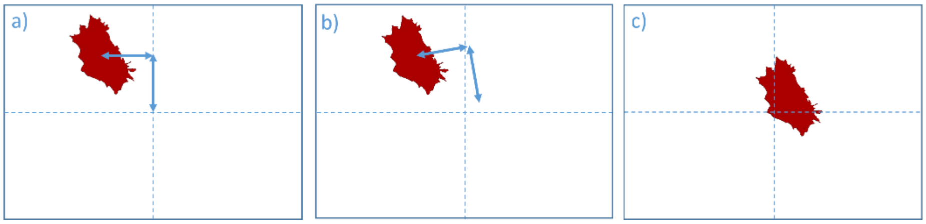

Figure 5: Effects of a camera rotation angle of 10 degrees: a) translation delta computed, b) effect of camera rotation angle on translation delta, c) translation delta applied producing non-centered colony

1.5 Generate test dataset

For each of the 3 reference plates (Day2, Day3, Day4), we acquired 5 sets of image tiles at varying overlap (Table 1). Each set of image tiles covers the same region on the plate as the reference measurements.

Table 1: Reference and evaluation dataset summary

Dataset acquisition	Overlap area as FOV percentage	Area covered (pixels)	Number of reference colonies
**Day2**	10, 20, 30, 40, 50	19500 x 16900	516
**Day3**	10, 20, 30, 40, 50	17300 x 17300	388
**Day4**	10, 20, 30, 40, 50	18300 x 17800	246

1.6 Evaluate Stitched Images

Stitching algorithm performance is evaluated by comparing colony measurements (count, centroid location and area) derived from the stitched image with the reference measurements. There are two major steps involved: (1) extract single stitched colonies and compute their relative locations and size, and (2) match colonies between the reference dataset and the test datasets.

1.6.1 Colony Extraction

After running the stitching algorithms on the test datasets we segment the stitched image. However, to account for sample photobleaching, we used an optimized threshold per dataset (Day and Overlap) to match the number of segmented colonies with the relevant reference dataset (Table 2). The thresholds were selected by minimizing the false positive and false negative counts that occur during colony matching. Since colony matching requires a stitched image, the naïve (stage coordinate based) stitching was used.

Table 2: Stitched Image Thresholds

Dataset	10% overlap	20% overlap	30% overlap	40% overlap	50% overlap
**Day2**	592	538	564	542	552
**Day3**	544	480	514	498	534
**Day4**	482	480	542	490	524

Each segmented colony is cropped out of the stitched image using its bounding box. The extracted colony images are then adjusted to move the colony centroid to the middle of the extracted image. This converts the stitched image into a series of individual images each containing a single colony

with its relative location P_m in the stitching image. Figure 6 summarizes this process, showing the conversion of the stitched image into a set of individual colony images.

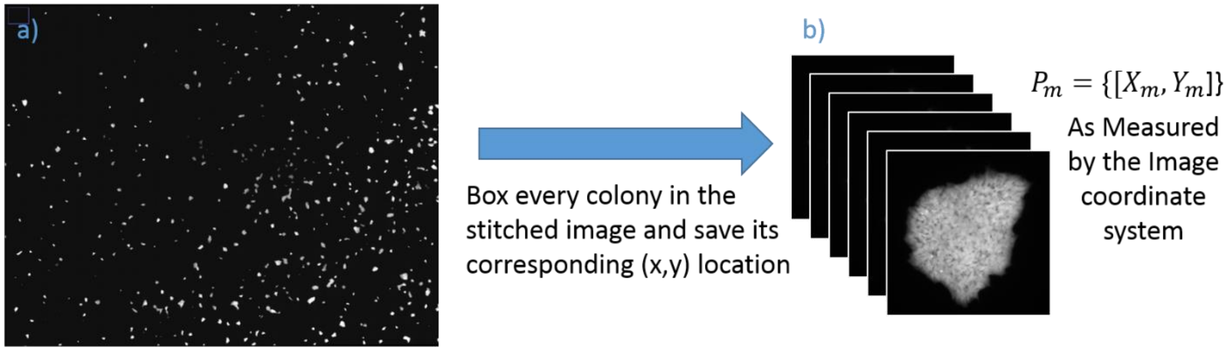

Figure 6: a) stitched image, b) colonies extracted with their relative locations

1.6.2 Colony Matching

We find an optimal match between the reference colonies and the colonies coming from the stitched image by computing a similarity matrix based on the Normalized Cross Correlation (ncc) in the range of -1 to 1 where a higher score correspond to better matching. The Hungarian algorithm [1] is then used to find an optimal matching between the reference and stitched colonies. Figure 7 summarizes the matching problem.

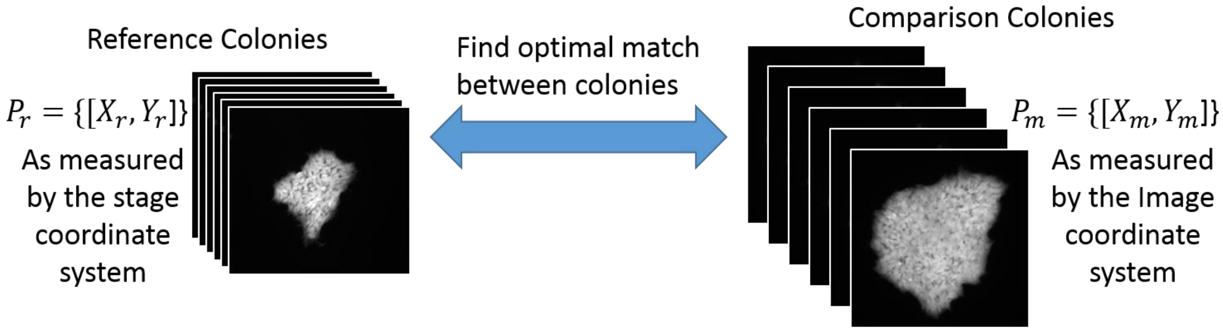

Figure 7: Matching between the reference colonies and the colonies from the stitched image

It is important to note that the centroid locations computed from the stitched image must be adjusted for rotation and translation due to the camera angle and the difference in location values between the absolute positions read on the microscope controller and the relative positions computed from the stitched image. We use the Kabsch algorithm [2] to compute the optimal translation matrix that minimizes the root mean squared deviation between the two sets as shown in Figure 8. However, we ignore the rotation component because the reference colony coordinates have already been rotated to match the camera angle (e.g., 0.0036 radians) as measured at the time of acquisition.

Algorithm: Kabsch

Input: Sets of points: X_{ref}, Y_{ref}, $X_{stitched}$, $Y_{stitched}$
Output: Translation Matrix T

```
begin
  P = [X_ref, Y_ref]          // P is a 2 × n_p matrix of n_p reference coordinates
  Q = [X_stitched, Y_stitched] // Q is a 2 × n_q matrix of n_q stitched coordinates
  Crop P and Q to the same size (2 × n)   // n is the number matching colonies
  P = P − P̄ where P̄_i = (Σ_{k=1}^{n} P_ik)/n           // Translate P to its centroid
  Q = Q − Q̄ where Q̄_i = (Σ_{k=1}^{n} Q_ik)/n           // Translate Q to its centroid
  H = PQ^T where H_ij = Σ_{k=1}^{n} P_ik Q_jk           // Compute covariance matrix
  H = BLW^T                 // Compute the singular value decomposition of H
  R = WB^T                              // Compute the optimal rotation matrix
  T = −RQ̄ + P̄                                        // Compute translations
  return T
end
```

Figure 8: Computing the optimal translation to minimize the root mean squared deviation between two points sets.

1.6.3 Stitching Performance Metrics

The stitching performance is assessed by computing the following 4 metrics; false positive, false negative, distance error, and size error. The output of a stitching method might result in adding some colonies by duplication or deleting some colonies by misaligning tiles. The result is a different number of reference and stitched colonies. Therefore, we label the number of reference colonies that are missing in the stitched image as False Negative (FN) and the number of colonies added in the stitched image but that do not exist in the reference dataset as False Positive (FP). The centroid distance error (D_{err}) is a global stitching accuracy measure and the colony size error (S_{err}) is a local stitching accuracy measure. The mathematical formulas for the two performance metrics are:

$$D_{err} = \left(\sum_{i=1}^{N} \sqrt{(x_{m_i} - x_{c_i})^2 + (y_{m_i} - y_{c_i})^2} \right) / N$$

$$S_{err} = \left(\sum_{i=1}^{N} \left| \frac{A_{c_i} - A_{m_i}}{A_{m_i}} \right| \right) / N$$

where D_{err} is the centroid distance error metric, (x_{m_i}, y_{m_i}) is the measured centroid location of reference colony i and (x_{c_i}, y_{c_i}) is its centroid location as computed from the stitched image. S_{err} is the size error metric; A_{m_i} is the measured area in pixels of reference colony i and A_{c_i} is the computed area from the stitched image and N is the number of reference colonies.

The size error (S_{err}) is predominantly affected by segmentation and not stitching. For each Day, the images were acquired in the following order: 10 %, 20 %, 30 %, 40 %, 50 %, and Reference. Photobleaching accumulates with repeated light exposure, so the reference images have the lowest fluorescent expression, as shown in Figure 9. The threshold selected for the Reference images results in additional pixels being considered foreground for the 10-50 % overlap images. This effect was minimized by dynamically selecting the threshold to minimize colony mismatch (see Section 1.6.1).

To quantify the error related to non-stitching factors, we computed S_{err} on a subset of stitching evaluation colonies known to be unaffected by stitching. We used only the stitching evaluation dataset colonies which fit completely within a single FOV (image tile) and are larger than 2100 pixels. The size threshold is 5 % higher than the regular minimum colony size defined in Section 1.3. This avoids small colonies that could have been pushed below the size threshold by photobleaching. Furthermore, during the matching phase (which relies on normalized cross correlation) only matches that are well correlated (correlation >0.9) were considered. Table 3 shows the resulting S_{err}. These values are similar to the results computed after stitching (presented in the main manuscript), they demonstrate that most of the size error comes from non-stitching sources (e.g., photobleaching). Figure 9 shows the change in colony foreground intensity due to photobleaching.

Table 3: Stitching S_{err} measurement results for colonies fully within a FOV

Metric	10% overlap	20% overlap	30% overlap	40% overlap	50% overlap
S_{err}	1 ± 6	1 ± 7	0 ± 7	0 ± 8	0 ± 8

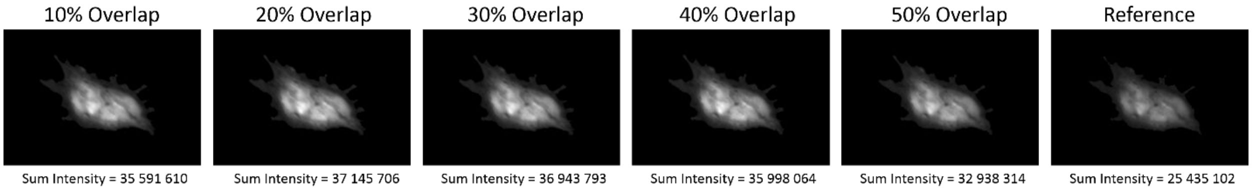

Figure 9: Change in colony intensity due to photobleaching with the sum intensity annotated.

1.7 Stitched Image D_{err} Heat Maps

To visualize the spatial distribution of the D_{err}, heat-maps were generated where the colony mask was colored based on its distance error. Figure 10 shows the stitched image distance error heat-maps for the Day4 10% overlap dataset. Most colonies are dark blue indicating a distance error of less than 10 µm. As the colonies get farther from the center of the image their distance error generally increases, showing up as cyan or green.

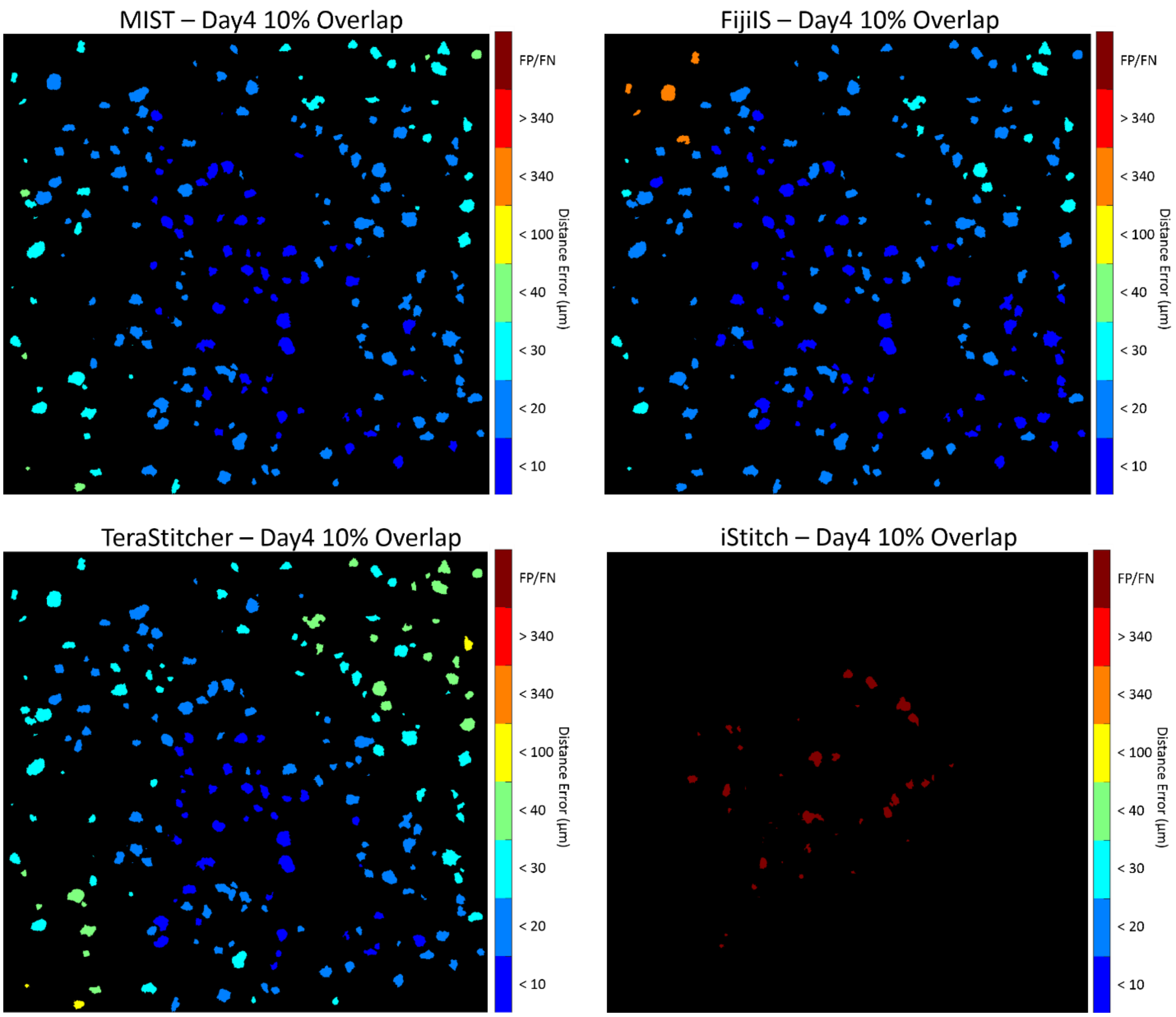

Figure 10: Day4 10% Overlap distance error heat-map

2 Implementation and Performance

All runtimes discussed here were generated using the following evaluation system. The reported runtimes are, unless otherwise specified, the average of 10 consecutive runs discarding times below the 10th percentile and above the 90th percentile to reduce potential measurement variability.

- Operating System: Ubuntu 14.04.4 LTS (Linux kernel 3.19.0-68)
- CPUs (2x):
    - Intel(R) Xeon(R) CPU E5-2650 v3 @ 2.30GHz (10 physical cores, 20 logical)
- Memory: 128GB DDR4
- GPUs (2x):
    - NVIDIA Tesla K40 (12GB on GPU memory)
- Disk: 7200 rpm SATA III hard disk
- SSD: SATA III Micron M600
- Open JDK 1.7
- CUDA Toolkit 7.5

2.1 Demonstration Datasets

The following datasets were used to demonstrate stitching on a variety of image types and content. Table 4 shows the image grid size, overlap between adjacent images, and resulting stitched image size from the examples shown in Figure 2 in the main document.

Table 4: Stitching Implementation Evaluation Datasets

Content Type	Grid Size	Overlap	Image Size (pixels)
**(1) A10 Cells**	42 x 59	75 %	17 300 x 22 800
**(2) Carbon Nanotubes**	20 x 30	10 %	55 000 x 55 000
**(3) HBMSC**	5 x 5	50 %	7100 x 7100
**(4) IPS Cells**	70 x 93	10 %	86 400 x 86 000
**(5) Paper Nanoparticle**	5 x 5	10 %	14 300 x 9500
**(6) Rat brain Cells**	8 x 5	8 %	10 300 x 4900
**(7) Stem Cells**	16 x 22	10 %	20 400 x 21 000
**(8) Worms**	14 x 19	10 %	17 700 x 17 900

A subset of these cropped zoomed example images are shown below to demonstrate the scale of the full stitched images: the A10 cells in Figure 11, the Carbon Nanotubes in Figure 12, and the Rat Brain cells in Figure 13.

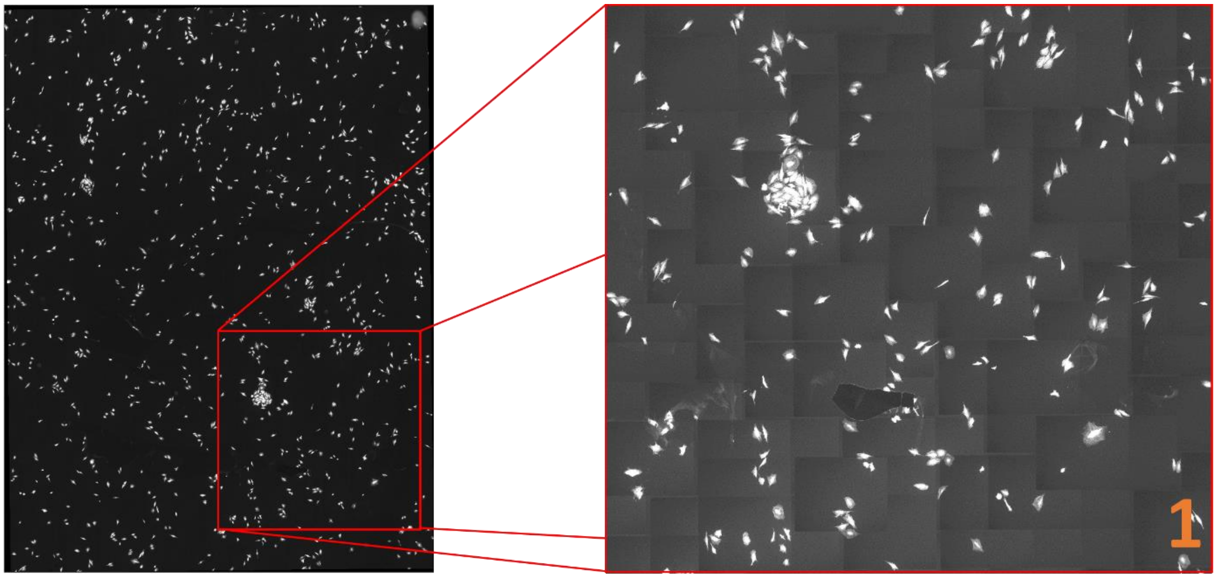

Figure 11: Example A10 Cells dataset showing zooming in sub-region (image contrasted adjusted for visual clarity).

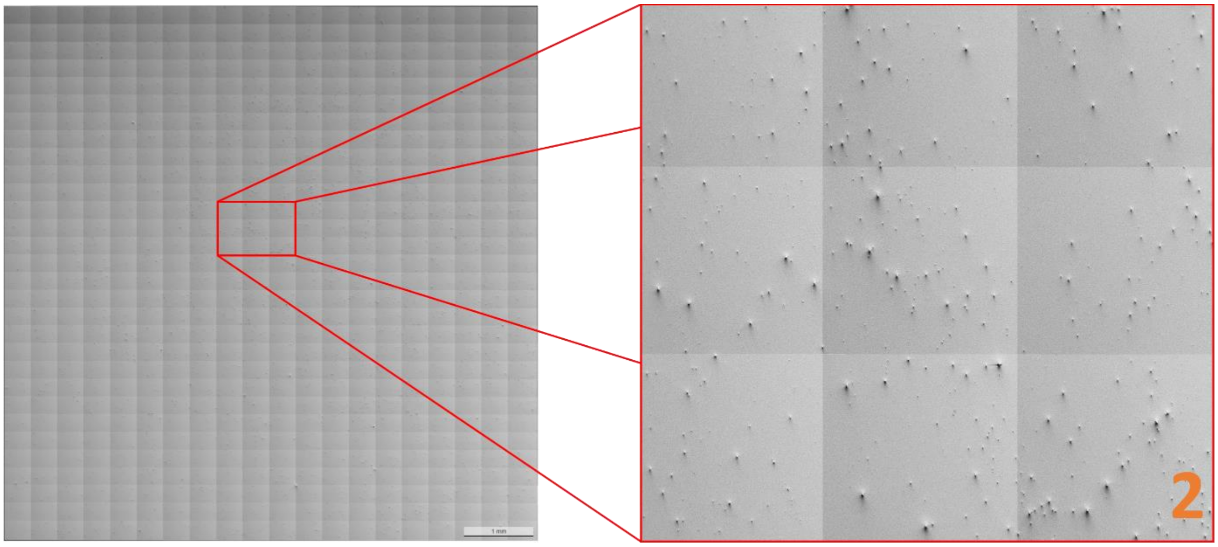

Figure 12: Example Carbon Nanotubes dataset showing zoomed in sub-region (image contrasted adjusted for visual clarity).

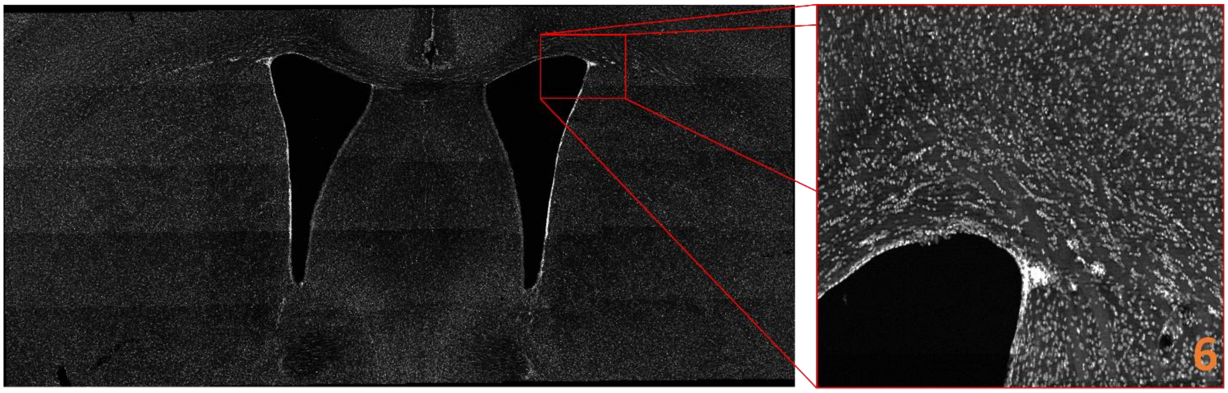

Figure 13: Example Rat Brain Cells dataset showing zoomed in sub-region (image contrasted adjusted for visual clarity).

2.2 Application Details

MIST was initially prototyped, designed, and validated in MATLAB. Once the MATLAB prototype was stable, MIST was ported to Java as an ImageJ/Fiji plugin for ease of use. By releasing MIST as a plugin within the ImageJ/Fiji platform it will likely see wider use than releasing a standalone tool. The ImageJ/Fiji implementation of MIST runs on the CPU and/or GPU.

There are three variants (execution types) of the MIST implementation:

1) Java: the pure Java implementation leverages the mines.edu toolkit [3] for 32 bit Fourier transforms during the translation computation step.
2) FFTW [4]: replaces the slower Java FFTs with compiled native libraries in order to perform 32 or 64 bit Fourier transforms during the pairwise translation computation step.
3) CUDA[5]–[8]: uses one or more GPUs on a system to perform the computationally intensive parts of the stitching algorithm for large datasets.

There are three major attributes that impact stitching runtime: (1) system memory, (2) number of CPU threads/number of cores, and (3) image storage location (network drive, hard drive, SSD, etc.). MIST is cognizant of the available system memory and scales the number of compute threads accordingly, which affects the amount of work that can be done in parallel. The performance of these threads is impacted by the time required to stream the input images to the computing hardware (i.e. reading from disk). This problem is further complicated when streaming to a GPU.

Our paper, Blattner et al [9], describes in detail the performance oriented software architecture including threading, memory management, application state management, and accelerators.

Please see our wiki for more details about the application and its use https://github.com/usnistgov/MIST/wiki.

2.3 Time-Lapse Application Dataset Performance

A primary motivation for developing MIST was the lack of stitching tools able to handle feature sparse image data. As the quantity of identifying features in the overlapping image area increases, the stitching task becomes easier as there is more information available for image stitching. Feature sparse image grids occur in the early time-points of long time-lapse image sequences of growing stem cells to give colonies room to grow.

To demonstrate the impact of this feature sparsity, we use the Stem Cell Replicate 1 time-lapse experiment imaged for a period of five days (available at https://isg.nist.gov/deepzoomweb/data/stemcellpluripotency). Each image grid in Replicate 1 consists of 18 x 22 phase-contrast images at 10% overlap. We stitched this dataset using all four methods. A full combinatorial exploration of the FijiIS parameter space was performed to determine that only the regression threshold (*rT*) affects the stitched image quality for this dataset. The default value of *rT* does not produce a viable stitched image for any time-point. As the *rT* value is lowered, FijiIS requires more runtime but produces more accurate stitched images. “If the regression threshold between two images after the individual stitching are below

that number [regression threshold] they are assumed to be non-overlapping" (see https://imagej.net/Image_Stitching under the "regression threshold" paragraph of the "Grid/Collection Stitching" section for more details about the FijiIS parameters). The rT parameter controls when two images are estimated to be non-overlapping. We speculate that by lowering the rT parameter, fewer pairwise translations are immediately discarded, thereby allowing the FijiIS translation optimization to eventually converge to a satisfactory answer. FijiIS uses rT values of 0.3 (default), 0.1, and 0.01. Any additional reduction in rT beyond 0.01 does not increase the stitched image accuracy of FijiIS. Finally, all stitching methods were limited to 10^4 seconds of runtime per time point, though only iStitch encountered that limit for a subset of the time points.

The general correctness of the stitched images is quantified using D_{err}, the average distance between each image tile's position in the stitched image and its position in an ideal image grid of exactly 10% overlap rotated to match the measured camera angle of 0.011 radians. To compensate for the lack of a shared coordinate system, the image tile locations are translated to minimize the least squares distance between the two sets of points. The average image tile D_{err} and required runtime are shown in Figure 4 in the main document for MIST, TeraStitcher, iStitch, and FijiIS.

As the time-lapse image sequence progresses and the images become more feature rich, the FijiIS stitching results improve in accuracy while requiring less runtime. Given that a microscope's Field of View (FOV) is 1040 x 1392 pixels, any D_{err} above 10^2 is roughly equivalent to 10% of a FOV and any D_{err} above 10^3 is roughly equivalent to a full FOV. To link D_{err} to a visual evaluation of the stitched images, three time points (10, 80, and 150 or the nearest time point with a stitched image) are shown in Figure 3 in the main document. To ensure that each image shares the same pixel size (scale) they have been padded to match the expected dimensions given the number of image tiles and the approximate overlap. With a $D_{err} \approx 10^2$ gaps can appear in the middle of the stitched image (main document Figure 3: Time Point 10 – FijiIS rT=0.01). These gaps were deemed unacceptable for quantifying the fluorescence expression of cell colonies. Despite FijiIS producing viable results (D_{err} = 32 pixels) for feature rich time points, the lack of reliability with feature sparse images and the runtime required (500-3000s per time point) makes FijiIS impractical for this type of application.

MIST was created to address these two concerns; (1) reliably stitching feature sparse microscopy images and (2) reducing compute time. If the stitching runtime is reduced to interactive rates, the user can efficiently interact with their data. Stitching the 161 StemCell-Replicate1 time points requires 73.7 hours with FijiIS at a rT of 0.01. MIST requires just 36.6 minutes using one GPU or 67.2 minutes using the two high-end Intel Xeon CPUs on our test system.

3 MIST Stitching Limitations

MIST has three noteworthy limitations.

3.1 A Regular Grid is Required

MIST was designed to stitch microscopy images acquired using a mechanical stage which can move the sample in a repeatable pattern. More specifically, MIST expects the overlap between

images to be approximately constant per direction (vertical and horizontal). For example, an image grid with an overlap of 10% ± 1% in the vertical direction and 13% ± 2% in the horizontal direction meets this requirement. Overlap uncertainties above 3% (the estimated actuator backlash) are generally unreliable.

More generally, if a collection of images to be stitched does not fit into our Stage Model (See Supplementary Document Section 4) MIST will not be able to correctly stitch the images.

3.2 Limited to 2D+time+channel Image Sequences

MIST is designed to perform 2D time-sequence multichannel stitching. It cannot perform volumetric stitching (3 spatial dimensions). However, MIST can stitch a numbered series of independent image grids. The nominal use case is stitching a time-series of grids. For this to work the images must be named so that the time slice is denoted as a number within the image filename. For example, the row-column filename pattern "img_<rrr>_<ccc>_<ttt>.tif" where the "<ttt>" is replaced with the current time slice number. This allows MIST to iterate over the time points, stitching each grid individually. This functionality can also be used to iteratively stitch Z slices of an image volume; however, this will treat each z-slice independently and no registration is performed between slices that are adjacent in the z-stack.

On the other hand, datasets that can use the same image positions, such as stitching multiple channels, can be stitched by MIST. Unlike time series, channels do not necessarily have an easy numerical representation which makes iterating over them difficult. Therefore, MIST was designed to allow the user to stitch a single channel and then apply those image positions to any other collection of images with the same grid dimensions. This functionality was used for our StemCell-Replica1 dataset where all 161 time points were stitched in the phase-contrast channel before assembling the fluorescent channel using the same image positions.

3.3 Noisy Images Might Require Preprocessing

MIST uses normalized cross correlation (ncc) as the metric of image similarity when performing registration. To refine translations between images, MIST performs a constrained hill climbing [10] with ncc as the cost function. Hill climbing searches the local neighborhood (valid translations 4-connected to the current translation) and selects the translation with the highest correlation. The algorithm terminates when a local maxima is found. Please see the technical algorithm documentation on the wiki (https://github.com/usnistgov/MIST/wiki) for more details about the translation refinement algorithm. However, in the presence of high noise levels this ncc surface has many local maxima, which can cause the hill climbing algorithm to converge on an incorrect local maximum, rather than the global maximum. A preprocessing step may be required to reduce the effects of noise. Figure 14 shows the hill climbing refinement for three pairs of images with different levels of noise.

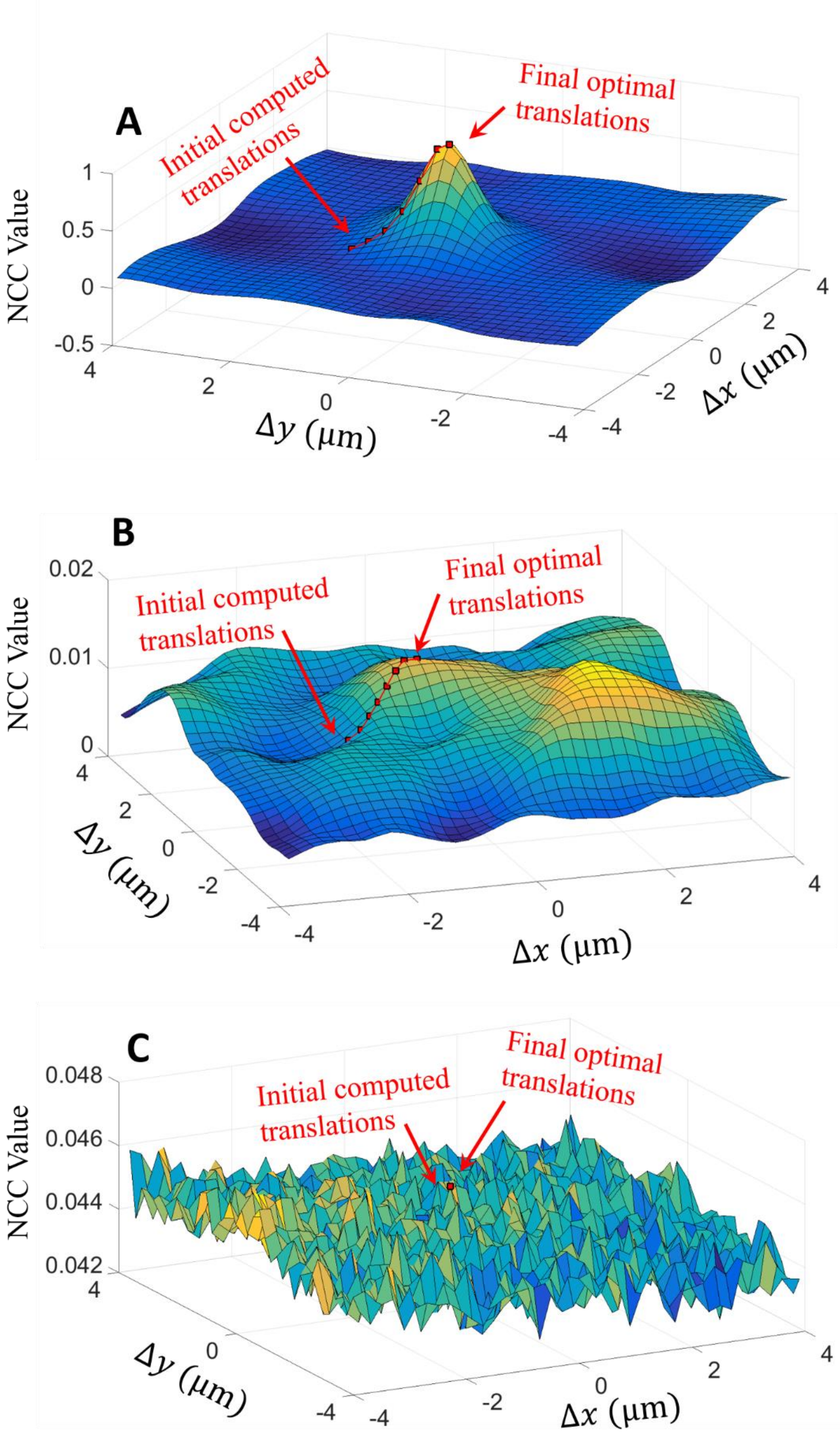

Figure 14: Normalized cross correlation (ncc) values between three pairs of adjacent images with a repeatability r=2μm: a) low noise level with a clear ncc peak, a) medium noise level with multiple local ncc peaks, and c) high noise level without a clear ncc peak. The red dots in each figure denote the path of the hill climbing algorithm.

The FIB-SEM image data is used to demonstrate the effect of noise on an application dataset. These images have a very low signal to noise ratio, contain prohibitive levels of image noise, and are very feature sparse. An example image is shown in Figure 15.

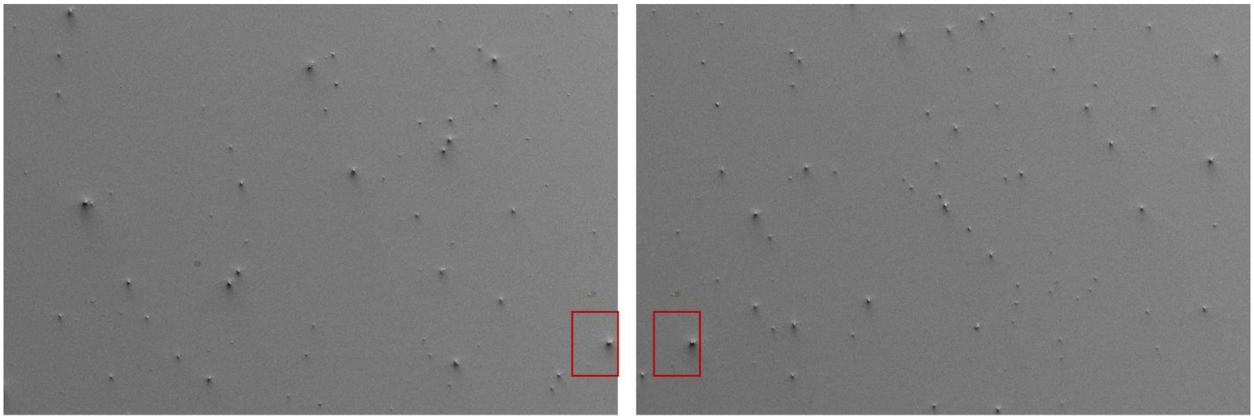

Figure 15: Example FIB-SEM consecutive image on the horizontal direction with 10% overlap, low SNR and sparse features. Inset rectangle denotes same sub-region in both images shown in Figure 16.

The red boxed region shown in Figure 15 belongs to two consecutive images on the horizontal direction with 10% overlap. Figure 16 demonstrates the smoothing effects on the ncc surface, computed between the left sub-region and the right sub-region, using different Gaussian filter sizes. Preprocessing the images helps reduce noise levels while smoothing the ncc surface, providing the hill climbing a better opportunity to find the correct peak. Figure 16 (a) shows a sub-region of both overlapping images as well as the ncc surface. The ncc surface is very noisy and finding the correct translation peak buried within this noise is difficult with hill climbing. The same procedure is repeated, except each image is filtered using two Gaussian filters: (b) $\sigma = 1$ and (c) $\sigma = 2$. The smoothing reveals a dominant peak, which hill climbing will converge to. Eventually, a large enough sigma for the Gaussian filter will blur out too many details and the stitching accuracy will degrade. For this dataset, a Gaussian filter with a sigma of 1 was sufficient to enable stitching.

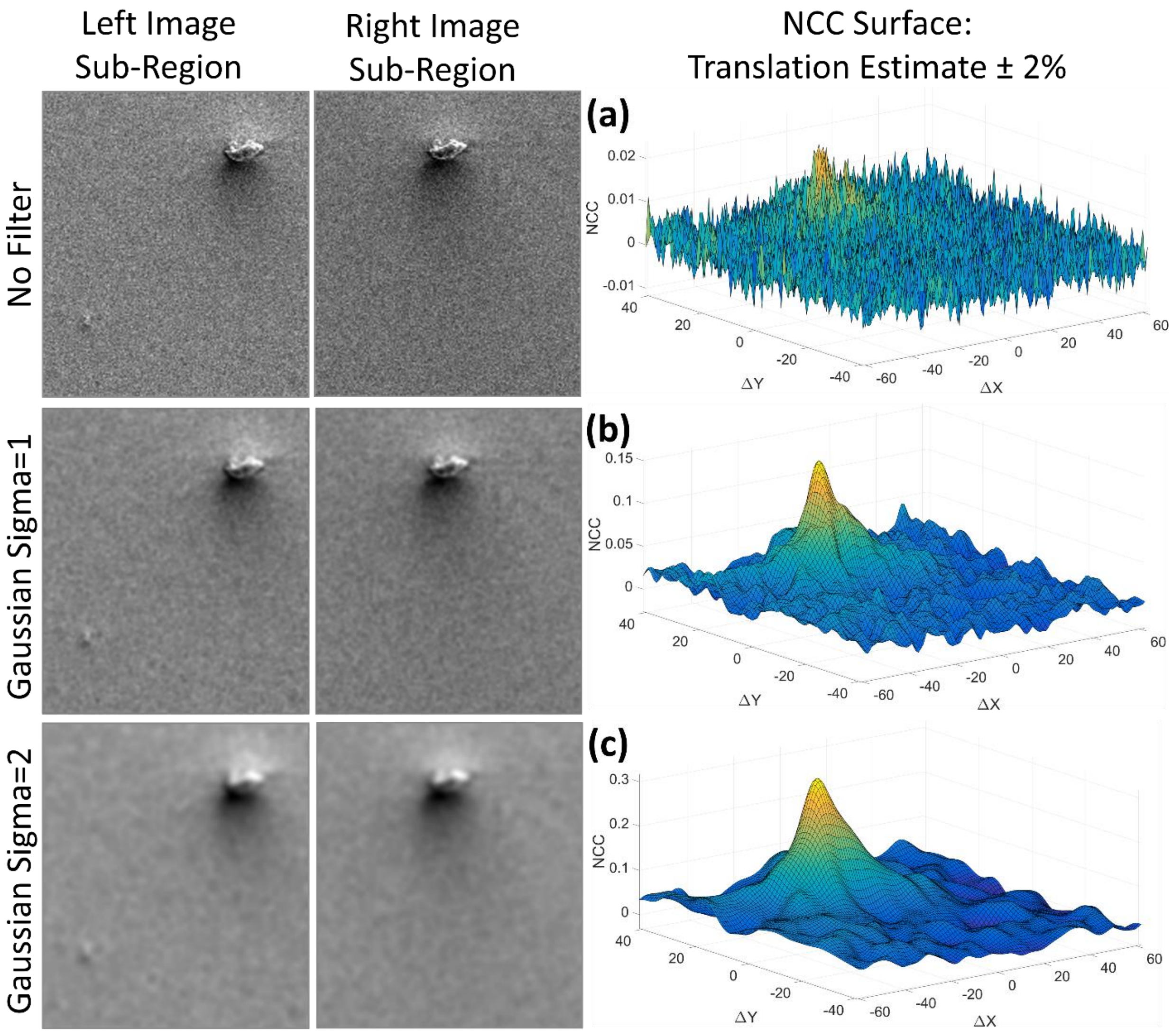

Figure 16: Normalized cross correlation surface response to gaussian filtering to reduce image noise. The left and right subregions are the same boxed region between two consecutive images on the horizontal direction with 10% overlap.

4 XY-Stage Actuator Movement Model

A motorized mechanical XY-Stage moves a biological sample with respect to the microscope's optical imaging system. This movement is carried out by two independent stepper motor linear actuators, one per direction. Therefore, it is important to understand the mechanical limitations of a stepper motor linear actuator mainly with regards to its accuracy and repeatability.

Accuracy: Actuator motion can only be as accurate as the mechanical components permit. The accuracy is the actuator's ability to provide precise increments of motion along its axis. In a mechanical system, this primarily concerns the lead screw as well as the feedback device (encoder, linear scale, etc.). Lead accuracy of the screw, resolution of encoders, and ability of the

controller must combine to produce the desired accuracy. In most microscopes, the accuracy is very high and can be calibrated.

Repeatability: Repeatability is the device's ability to reach a specific location repeatedly. It does not consider the accuracy of the position but rather the ability to go back to the same position. Many times, the actuator will follow a slightly bowed or twisted path due to imperfect construction. The repeatability is the measure of uncertainty relative to a given actuator movement. The repeatability cannot be calibrated, but it can be measured for any microscope stage.

In a grid tiling, the positions (x, y), that the stage will go to, have an uncertainty equal to the stage repeatability $(\mathrm{x} \pm \mathrm{r}_x, \mathrm{y} \pm \mathrm{r}_y)$. However, translations $(\mathrm{dx}, \mathrm{dy})$ computed in the vertical or horizontal directions between consecutive tiles are differences between respective positions. Thus, the uncertainty on the computed translation values is $2\times$repeatability $(\mathrm{dx} \pm 2\mathrm{r}_x, \mathrm{dy} \pm 2\mathrm{r}_y)$. The actuators are independent from one another. However, since they are very similar and often from the same manufacturer, we will assume that $\mathrm{r}_x = \mathrm{r}_y = \mathrm{r}$.

When computing vertical translations between two consecutive tiles, the dx and dy components will correspond to the projection of the stage displacement on the camera coordinate systems (which may have a fixed rotational angle) with an uncertainty equal to $2\times$repeatability. We use this information to estimate the stage repeatability from the computed translations.

5 Stage Model Parameter Estimation

Our stage model leverages knowledge of how a motorized XY-Stage operates to introduce constraints on the translations. The stage model has four parameters: (1) image overlap amount, (2) camera angle, (3) the microscope stage repeatability, and (4) the microscope stage backlash.

The image overlap is estimated per direction by fitting a model to the translations using maximum likelihood estimation. The model expects that the translations can be split into two subsets; a set of normally distributed valid translations and a uniformly distributed set of invalid translations. This model is described by three parameters: the mean (μ) and standard deviation (σ) of the normal distribution, and the probability of a translation belonging to the uniform distribution (p). The likelihood of any given model (μ, σ, p) for a set of N translations $(t_1, t_2, \dots, t_N)$ is given by the following equation:

$$likelihood_{\mu,\sigma,p} = \sum_{i=1}^{N} \ln\left(\left| p\times\frac{1}{t_{range}} + (1-p)\times\left(\frac{1}{\sigma\sqrt{2\pi}}\exp\left(-\frac{(t_i-\mu)^2}{2\sigma^2}\right)\right)\right|\right)$$

where the range of possible translations for that direction is given by t_{range}. By finding the model which maximizes this likelihood, an estimation of the overlap between images can be extracted from the average valid translation.

The stage repeatability is estimated by heuristically filtering the translations per direction and looking at the range of valid translation values. For each translation direction, the x & y

repeatability metrics are computed independently. The maximum of all the computed repeatability values is used to constrain the hill climbing translation optimization.

Please see the technical algorithm documentation on the wiki (https://github.com/usnistgov/MIST/wiki) for the specific implementation details of the estimation of the stage model parameters.

6 Tilt and plate movement Assessment

We analyzed the tilt and the displacement of the plate as the stage moved as well as when a biologist must take out the plate for feeding living cells every 24 h. During this live cell experiment, the biologist had to physically take out the plate and change the solution with added nutrients, place the plate back on the stage and re-center the plate every 24 h.

To quantify the effect of the tilt and plate movement, we tested the procedure using a plate with added fiduciary markers (‘X’ and dots placed on the plate itself). We imaged the plate and computed the centroid locations of all fiduciary markers and some cell colonies. We then followed the procedure of feeding the cells (placed the plate back on the stage and centered the stage using the microscope controller). We then visited all previously registered fiduciary markers and cell colonies centroid locations. They were all within the repeatability limit of the microscope.

7 Image Composition

To perform mosaic image composition, each adjacent pair of images must have a relative translation (displacement). These translations form an over-constrained system that one can represent as a directed acyclic graph where vertices are images and edges are weighted based on the correlation between adjacent images. The over-constraint in the system is due to the equivalence between absolute displacements of images and path summations in the graph; these summations must be path invariant to yield a well-formed image. In general, this phase resolves the over-constraint in the system and computes absolute displacements. This is achieved by selecting a subset of the relative displacements [11]–[13] or applying a global optimization approach to adjust them to a path invariant state [14]. These absolute displacements are used to compose the stitched mosaic image.

Each image (vertex) has translations relative to (up to) 4 neighbors. Some of these translations may be conflicting. MIST resolves this over-constraint conflict by selecting a subset of the relative displacements resulting in a path invariant and well-formed image. This is achieved using a maximum spanning tree with the translation normalized cross correlation values as the undirected graph edge weights. Edges are selected to maximize the sum of all selected edge weights such that each image tile is connected once and only once. We use Prim’s weighted maximum spanning tree algorithm to perform this selection [15]. The edge weights from valid translations are promoted with a constant value to ensure they are always used before a non-valid (estimated) translation when determining the spanning tree. A maximum spanning tree is used to maximize the set of edge weights used in assembling the tree because, the higher the edge weight, the higher the confidence in the translation associated with the particular edge.

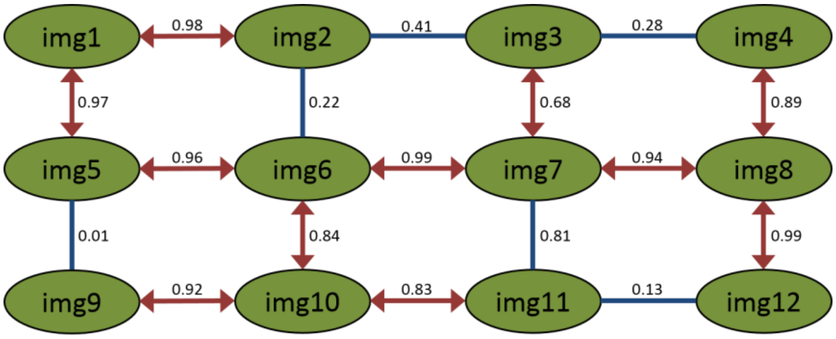

Figure 17: Image mosaic assembly represented as an undirected graph with tiles considered as nodes and translations (ncc values) as edges. Prim's weighted maximum spanning tree algorithm is used to find the subset of edges to connect all image tiles to form the reconstructed stitched image. Each edge shows its ncc weight. Selected edges are displayed in red, non-selected edges in blue.

With the absolute displacements computed, each image tile in the Image Grid has a global (x, y) location in the stitched mosaic image. The stitched image is built by copying the individual image tiles into position within the stitched image and blending the image tiles together. Three types of blending are implemented: overlay blending places the image with the higher correlation on top, average blending averages all overlapping pixels together, and linear blending weights the pixels based on the distance to the edge of the component image tile [14].

8 References

[1] J. Munkres, "Algorithms for the Assignment and Transportation Problems," *J. Soc. Ind. Appl. Math.*, vol. 5, no. 1, pp. 32–38, 1957.

[2] W. Kabsch, "A discussion of the solution for the best rotation to relate two sets of vectors," *Acta Crystallogr. Sect. A*, vol. 34, no. 5, pp. 827–828, 1978.

[3] D. Hale, "Mines Java toolkit: Java packages for scientific computing," 2014. [Online]. Available: http://inside.mines.edu/~dhale/jtk/.

[4] M. Frigo and S. Johnson, "The Design and Implementation of FFTW3," *Proc. IEEE*, vol. 93, no. 2, pp. 216–231, 2005.

[5] NVIDIA, "cuFFT Library," 2014. [Online]. Available: https://developer.nvidia.com/cuFFT. [Accessed: 30-Jun-2015].

[6] NVIDIA, "CUDA Toolkit," 2014. [Online]. Available: https://developer.nvidia.com/cuda-toolkit. [Accessed: 30-Jun-2015].

[7] NVIDIA, "JCuda." [Online]. Available: http://www.jcuda.org/jcuda/JCuda.html. [Accessed: 30-Jun-2015].

[8] NVIDIA, "JCufft." [Online]. Available: http://www.jcuda.org/jcuda/jcufft/JCufft.html. [Accessed: 30-Jun-2015].

[9] T. Blattner, W. Keyrouz, J. Chalfoun, B. Stivalet, M. Brady, and S. Zhou, "A Hybrid CPU-GPU System for Stitching of Large Scale Optical Microscopy Images," in *43rd International Conference on Parallel Processing (ICPP)*, 2014, pp. 9–12.

[10] S. Russell and P. Norvig, *Artificial Intelligence: A Modern Approach*, vol. 2. 2003.

[11] A. Bria and G. Iannello, “TeraStitcher - A tool for fast automatic 3D-stitching of teravoxel-sized microscopy images.,” *BMC Bioinformatics*, vol. 13, p. 316, 2012.

[12] Y. Yu and H. Peng, “Automated high speed stitching of large 3D microscopic images,” *2011 IEEE Int. Symp. Biomed. Imaging From Nano to Macro*, pp. 238–241, Mar. 2011.

[13] A. Bria, L. Silvestri, L. Sacconi, F. Pavone, and G. Iannello, “Stitching terabyte-sized 3D images acquired in confocal ultramicroscopy,” *Proc. - Int. Symp. Biomed. Imaging*, pp. 1659–1662, 2012.

[14] S. Preibisch and S. Saalfeld, “Fast stitching of large 3d biological datasets,” *Proc. ImageJ User Dev. Conf.*, 2008.

[15] R. C. Prim, “Shortest Connection Networks And Some Generalizations,” *Bell Syst. Tech. J.*, vol. 36, no. 6, pp. 1389–1401, 1957.
